# Chiropractic spinal manipulative therapy for acute neck pain: A 4-arm clinical placebo randomized controlled trial. A prospective study protocol

**DOI:** 10.1371/journal.pone.0295115

**Published:** 2023-12-07

**Authors:** Aleksander Chaibi, Anna Allen-Unhammer, Nina Køpke Vøllestad, Michael Bjørn Russell

**Affiliations:** 1 Department for Interdisciplinary Health Sciences, Institute of Health and Society, Faculty of Medicine, University of Oslo, Oslo, Norway; 2 Head and Neck Research Group, Division for Research and Innovation, Akershus University Hospital, Lørenskog, Oslo, Norway; 3 Campus Akershus University Hospital, Institute of Clinical Medicine, University of Oslo, Nordbyhagen, Norway; University of Southern Denmark, DENMARK

## Abstract

**Introduction:**

Neck pain poses enormous individual and societal costs worldwide. Spinal manipulative therapy and **N**on-**S**teroidal **A**nti-**I**nflammatory **D**rug treatment are frequently used despite a lack of compelling efficacy data. This protocol describes a multicentre 4-arm, clinical placebo randomized controlled trial (RCT), investigating the efficacy of chiropractic spinal manipulative therapy (CSMT) versus sham CSMT, ibuprofen, and placebo medicine for acute neck pain. This superiority study will employ parallel groups, featuring a 1:1:1:1 allocation ratio.

**Material and methods:**

We will randomize 320 participants equally into four groups: CSMT, sham CSMT, ibuprofen, or placebo medicine. CSMT groups are single-blinded, while the medicine groups are double-blinded. Data will be collected at baseline (Day 0), during treatment and post-treatment. The primary endpoint will assess the difference in mean pain intensity from Day 0 to Day 14 on a numeric rating scale 0–10; the CSMT group is compared to sham CSMT, ibuprofen, and placebo medicine groups, respectively. Secondary endpoints will assess mean pain intensity and mean duration at different time points, and adverse events, blinding success, and treatment satisfaction, including comparison between ibuprofen and placebo medicine. Power calculation is based on a mean neck pain rating of 5 at Day 0, with standard deviation of 1 in all groups. Mean pain reduction at Day 14 is expected to be 60% in the CSMT group, 40% in sham CSMT and ibuprofen groups, and 20% in the placebo medicine group. A linear mixed model will compare the mean values for groups with corresponding 95% confidence intervals. P values below 0.017 will be considered statistically significant. All analyses will be conducted blinded from group allocation.

**Discussion:**

This RCT aims towards the highest research standards possible for manual-therapy RCTs owing to its two placebo arms. If CSMT and/or ibuprofen proves to be effective, it will provide evidence-based support for CSMT and/or ibuprofen for acute neck pain.

**Trial registration:**

ClinicalTrials.gov identifier: NCT05374057. EU Clinical Trials Register: EudraCT number: 2021-005483-21.

## Introduction

The Global Burden of Disease ranks musculoskeletal neck pain as the fourth most common disability worldwide [1]. The age-standardised prevalence rate for neck pain was reported to be 27.0 per 1000 population in 2019 [2], while the mean lifetime prevalence of neck pain is 48% (range 14–71%) [3]. Most instances of radicular or non-radicular acute neck pain resolve within three months, however, a substantial proportion of patients continue to experience low-grade symptoms or frequent recurrences [4, 5]. The financial burden of neck pain is unknown, but the combined cost of neck and lower back pain, is 87.6 billion USD annually in the United States [6–8]. Despite this, neck pain research is surprisingly meagre, and far less abundant than research on lower back pain [9].

After lower back pain, neck pain is the second most common complaint treated by chiropractors [10, 11]. Spinal manipulative therapy (SMT) is one commonly adopted non-pharmacological treatment alternative to Non-Steroidal Anti-Inflammatory Drugs (NSAIDs) for neck pain [12]. SMT is defined as a passively controlled manoeuvre that uses a directional high-velocity low-amplitude thrust directed at a specific joint, past the physiological range of motion, without exceeding the anatomical limit [13]. It is hypothesized that SMT may relieve neck pain by stimulating neural inhibitory systems at different spinal cord levels, through the activation of various central descending inhibitory pathways [14–24], but the physiological mechanisms of pain relief are not fully understood [25].

NSAIDs are among the most commonly used painkillers, yet compelling efficacy data on their use for acute neck pain is lacking [4, 11, 26, 27]. NSAIDs exhibit frequent and continuous side effects [28, 29], and are contraindicated in patients with gastric ulcers, heart failure (New York Heart Association (NYHA) class IV) or kidney failure (glomeruli infiltration <30 ml/min), while 3^rd^ trimester pregnant women should also avoid NSAIDs [30]. Some patients experience insufficient effect from NSAIDs, while other patients might avoid medication for various reasons [12, 26].

A recent systematic review and meta-analysis reporting on the effectiveness of SMT in the treatment of acute neck pain concluded that SMT alone, or in combination with other modalities is likely to be effective [12]. However, the authors emphasized that the results of the six included randomized controlled trials (RCTs) should be interpreted with caution, due to heterogeneity, small sample size, and unanswered questions regarding the placebo effect due to lack of blinding [12]. Thus, the need to identify safe and efficacious treatment(s) for acute neck pain as well as prioritize this field of research is highly evident.

This prospective clinical multicentre 4-arm chiropractic spinal manipulative therapy (CSMT) RCT will evaluate the efficacy and safety of CSMT and ibuprofen in the treatment of acute neck pain. Our primary hypothesis is that the CSMT group will experience a greater reduction in mean pain intensity from the inclusion point at baseline (Day 0) to end of treatment (Day 14), compared to the sham CSMT, ibuprofen, and placebo medicine groups. The null hypothesis is that there will be no difference in mean pain intensity change from Day 0 to end of treatment, between the four groups. This superiority study will employ parallel groups, featuring a 1:1:1:1 allocation ratio. The study is designed to avoid/minimize shortcomings and biases of previous manual therapy RCTs.

## Materials and methods

### Design and setting

**Fig 1:**
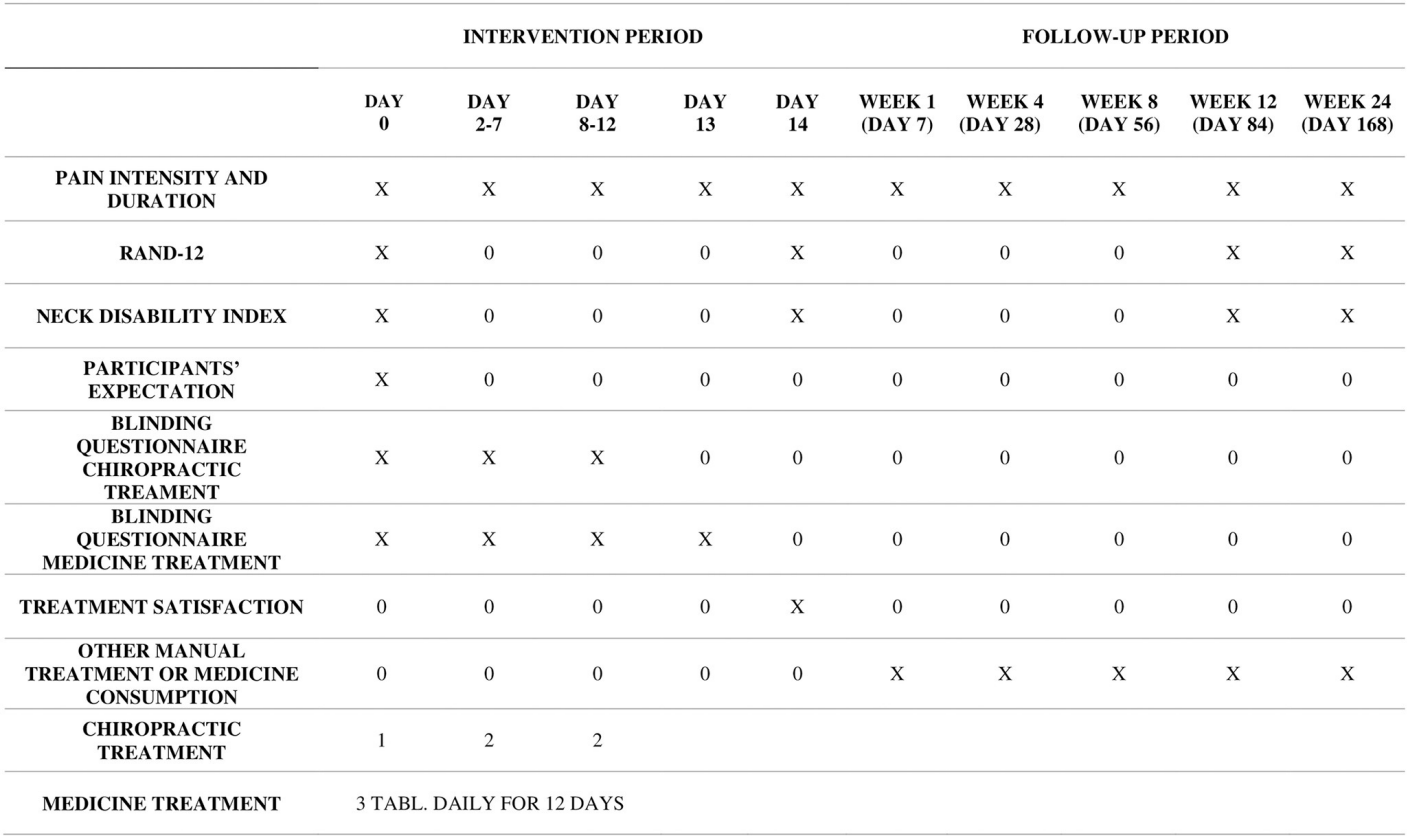
SPIRIT schedule. Data collection and treatment. Chiropractic treatment is either real or sham, while medicine is either ibuprofen or placebo medicine.

This study protocol is for a prospective clinical multicentre, 4-arm placebo RCT of 26 weeks’ duration; inclusion (Day 0), 2 weeks of treatment and 24 weeks follow-up (Fig 1).

We intend to invite 40 chiropractor investigators with at least 5-years of clinical experience and who are exclusively chiropractors. They work in Norwegian chiropractic clinics in major cities and are equally distributed by location and gender. The chiropractor investigators will recruit and treat enrolled participants.

**Fig 2:**
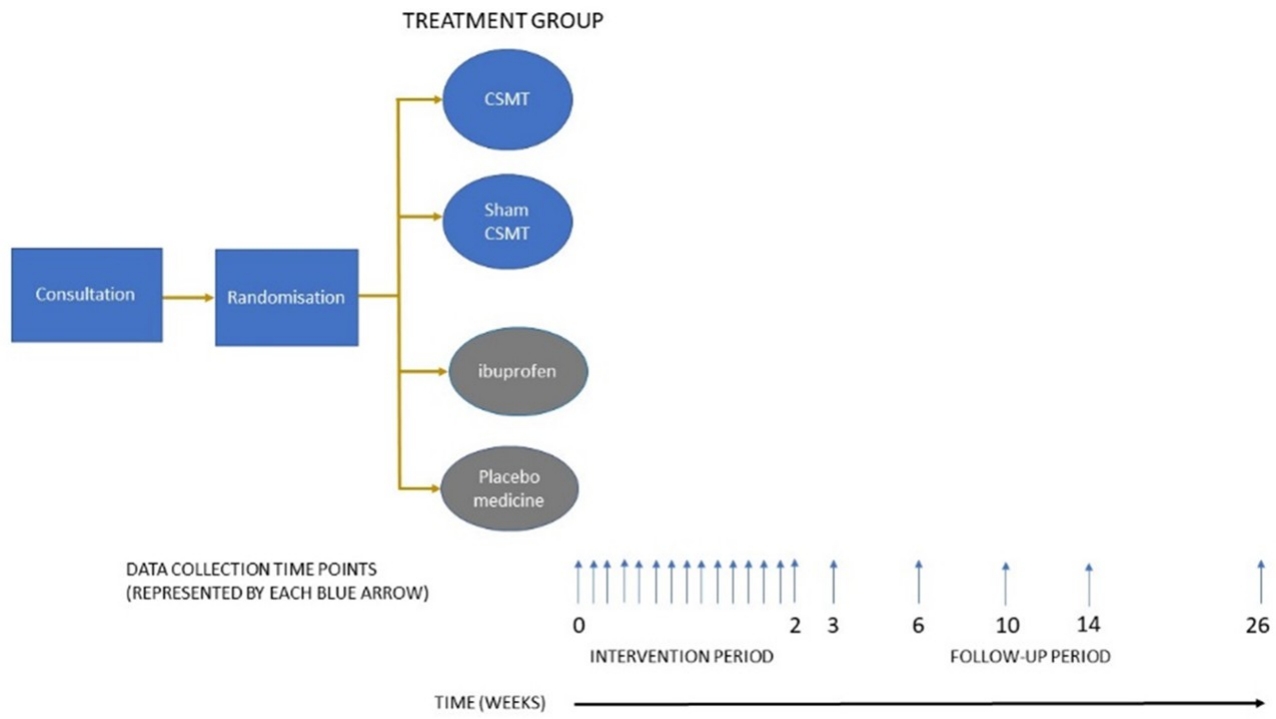
Study design. Chiropractic spinal manipulative therapy (CSMT).

**Fig 3:**
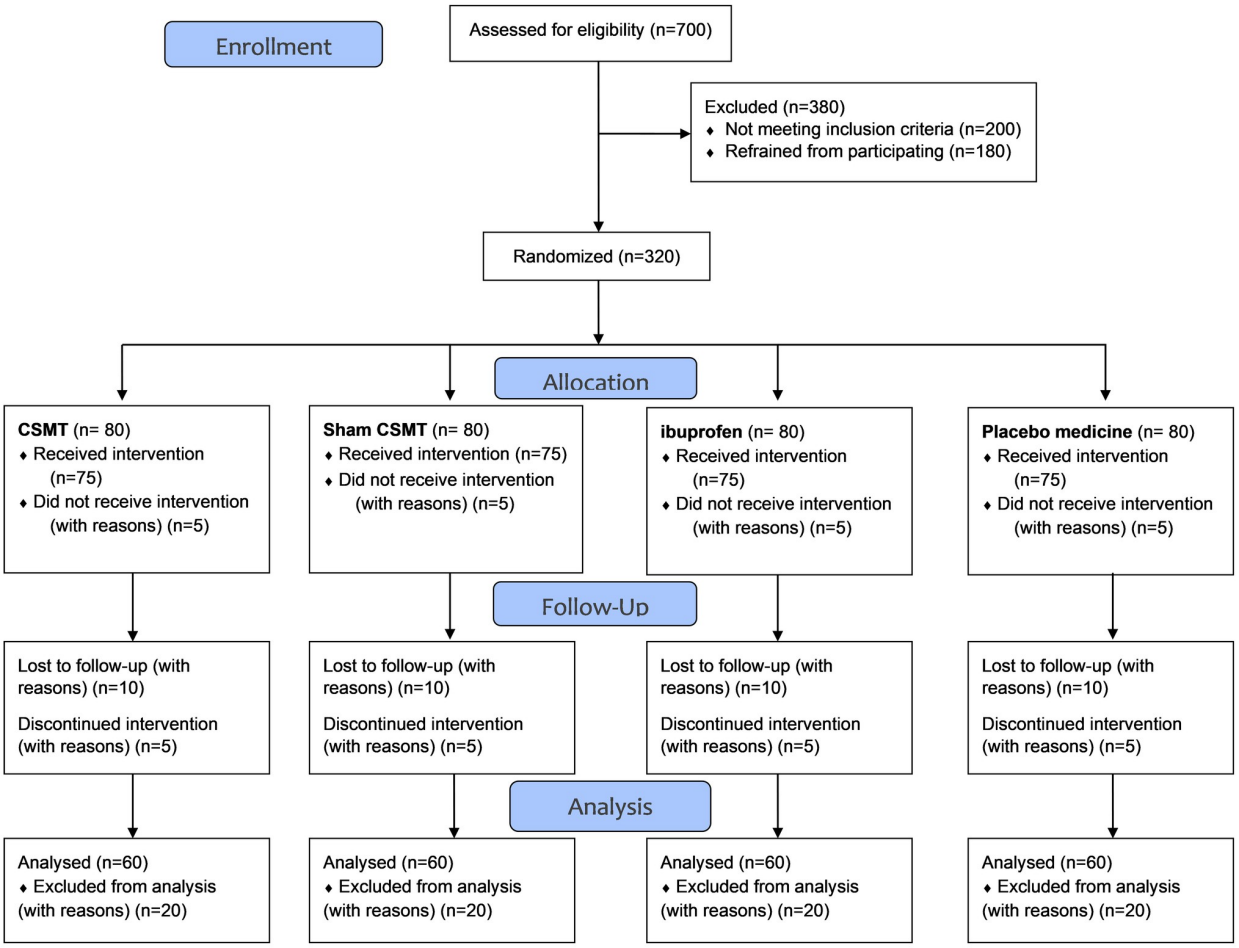
Expected participant flow diagram. Chiropractic spinal manipulative therapy (CSMT).

We aim to include 320 participants (Figs 2 and 3). Participants will receive an assessment and treatments free of charge, as an incentive to participate in the study.

This study will adhere to the Standard Protocol Items: Recommendations for Interventional Trials (SPIRIT) and CONSORT guidelines [31, 32].

The study has been registered at the Norwegian Medicines Agency (Statens Legemiddelverk), the EU Clinical Trials Register (EudraCT number: 2021-005483-21), ClinicalTrials.gov (Identifier: NCT05374057), and the Open Science Framework DOI: 10.17605/OSF.IO/AF49C.

### Randomization

The group allocation sequence in blocks of four represents each of the four treatments: CSMT, sham CSMT, ibuprofen or placebo medicine, and the continuous unique numeric participation ID-numbers are computer-generated randomly in a 1:1:1:1 allocation ratio. The randomization log will be prepared by an external party to ensure concealment from the research group. The log will be stored on a secure password protected database, inaccessible to the research group.

The chiropractor investigators will keep a secure list of participant names, mobile phone numbers and e-mail addresses, as well as ID-numbers in their local Trial Master File. The PhD student (author AAU) will have access to this list, to send the digital questionnaires via e-mail to the participants at various time points during the study.

### Chiropractor investigators and participants

Prior to inclusion of participants at Day 0, all chiropractor investigators have received verbal information about the clinical trial, a practical clinical demonstration of the Sharp-Purser test, and sham CSMT via a Zoom workshop, hosted by the research group (authors of this paper). The Sharp-Purser test is selected as part of the trial’s eligibility criteria with the purpose of screening for atlantoaxial instability. While it cannot be seen as a definitive stand-alone test, it may be clinically useful in combination with the experienced recruiting chiropractors’ clinical judgement, in the absence of a better alternative [33, 34].

The chiropractor investigators have also received an instruction video illustrating the different steps of the sham CSMT protocol.

Participants will be recruited from May 2022 to 31.12.2023. The chiropractor investigator or receptionist at each chiropractic clinic will pre-screen potential participants via telephone. The initial pre-screening criteria are acute neck pain within the previous 14 days; no chiropractic treatment received during the past 3 months, and age between 18 and 59 years. Those who fulfil the pre-screening criteria will be booked in for a full screening evaluation by the chiropractor investigator.

The chiropractor investigator will provide the potential participant with oral and written information about the project. Those whom wish to participate are checked against a list of inclusion (Table 1) and exclusion criteria (Table 2), and will receive a thorough clinical examination, including the Sharp-Purser test [35]. Participants that meet the inclusion criteria, and who do not violate the exclusion criteria, will be eligible for participation.

**Table 1:** Inclusion criteria.

Eligible participants are between the age of 18 and 59 years old Acute non-radicular neck pain, i.e., Grade 1 or 2 according to the classification by the Bone and Joint Decade 2000–2010 Task Force on neck pain [36] Onset of the present episode ≤ 2 weeks prior to the 1^st^ chiropractic visit Moderate, severe, or very severe pain intensity, i.e., ≥4, on a numerical rating scale (NRS) 0–10 Pain free for at least four consecutive weeks prior to the present pain episode Not treated by a chiropractor during the past 3 months Participant must accept not to seek other manual and/or pharmacological treatments for their acute neck pain during the intervention period Non-pregnant women. Women in doubt shall have a negative fertility test before inclusion

**Table 2:** Exclusion criteria.

Contraindication to ibuprofen active peptic ulcer gastrointestinal bleeding previous repeated episode (≥2 detected events) with peptic ulcer or gastrointestinal bleeding Previous gastrointestinal bleeding or ulcer using NSAIDs Hypersensitivity to ibuprofen Asthma induced by acetylsalicylic acid or other NSAIDs Urticaria Rhinitis Severe heart failure (New York Heart Association (NYHA) class IV) Renal failure (glomerulus infusion <30 ml/min) Taken pain- and/or anti-inflammatory medicine during the past 24 hours? (Patients that have taken acute pain- and/or anti-inflammatory medicine, including ibuprofen, can be included if they return after 24 hours without having taken the medicine where they then fill out questionnaires and are randomized at the clinic) On prescribed antidepressant Major psychiatric disorder Pregnancy or intention to be pregnant Contraindication to SMT Signs of spinal radiculopathy including progressive neurological deficit Upper cervical spine instability (positive Sharp-Purser test) [35] Previous fracture in the cervical and/or thoracic spine Previous cervical spine surgery Recent (<6 months) severe physical trauma to the head, neck, or thoracic spine Concomitant low back pain with moderate, severe, or very severe pain intensity (≥4 on an NRS) Current chronic pain (defined as ≥3 months duration) Rheumatoid arthritis Recent (<2 weeks) acute respiratory infection with fever Any presence of ischemic symptoms upon examination Horner’s syndrome Medical history of arterial anomalies History of connective tissue disorder Familial history of cervical artery dissection Other vascular disorders [37] Inability to understand instructions given in the Norwegian language Inability to fill out digital questionnaires Other reasons to exclude the patient as deemed necessary by the chiropractor

Participants will provide oral and written Informed Consent by filling in the baseline questionnaire at Day 0, which also includes a digital copy of the consent form. The following steps are performed during the first consultation at the chiropractor investigator’s office.

The participant draws an envelope revealing the participant’s unique 5-digit ID-number but does not yet open the sealed envelope that conceals group allocation. The participant then scans QR-code one with their mobile phone to access the baseline questionnaire. They fill in their 5-digit ID number and reply to questions in the waiting room.

Once completed, the participant re-enters the treatment room, and opens the envelope to reveal the treatment allocation, i.e., chiropractic manual therapy or medicine.

The chiropractor investigators are provided with separate randomization lists that reveal the manual treatment he/she should apply to the participant, i.e., CSMT or sham CSMT.

The participant then scans QR-code two and fills in a short questionnaire on treatment expectation in the waiting room. The chiropractor investigator receives an e-mail link and fills in a similar questionnaire on treatment expectation simultaneously in the treatment room.

Finally, the chiropractor investigator delivers the treatment according to the allocated group.

### Interventions and visits for participants

Active manual treatment will consist of CSMT [13], a specific contact, high-velocity, low-amplitude, spinal thrust manipulation directed to spinal biomechanical dysfunction in the cervical and/or thoracic spinal column, past the physiological range of motion, but without exceeding the anatomical limit, as diagnosed by standard chiropractic tests, in accordance with the chiropractor investigators’ clinical judgment. Participants in this group will receive five intervention sessions over 10–12 days, more specifically, one session at Day 0, two sessions during Days 2–7 and two sessions during Days 8–12.

Sham CSMT will consist of a broad, non-specific contact, low-velocity, and low-amplitude sham push manoeuvre in a non-intentional, non-therapeutic directional line [38–40]. All of the non-therapeutic contacts will be performed outside of the spinal column with adequate joint slack and without soft tissue pre-tension, so that joint cavitations do not occur and possible spinal cord afferent input is minimized [39]. The exact sham CSMT procedure is reported elsewhere [38].

Participants in the CSMT group whose spinal function is considered normalised upon physical assessment will not receive CSMT but will attend all five-intervention sessions and have their joint function evaluated.

Participants randomized to ibuprofen or placebo medicine will receive a package of 36 tablets, containing 600 mg ibuprofen or placebo medicine, to be taken three times per day for 12 days [41, 42]. The participants will acquire the medicine at the chiropractic clinic.

No additional co-interventions or specific advice apart from reassurance of “business as usual” and encouragement of normal activities will be given to all four groups during the trial period, in accordance with practice guidelines.

Participants in all four groups who do not experience spontaneous improvement, will after 6-months follow-up, be offered a free of charge chiropractic assessment and treatment for up to five treatment sessions, on the premises of the trial.

### Data collection

Data will be collected at Day 0, daily during the 2-week treatment period and 1-, 4-, 8-, 12-, and 24-weeks post-treatment. Demographic data on all investigators will be collected and descriptively presented. Fig 1. shows the SPIRIT schedule of data collection and treatment interventions. The specific wording of the neck pain questions is:

1. On average, how intense was your neck pain today, on a numeric rating scale (NRS) from 0 to 10, where 0 is no pain and 10 is unbearable pain?
2. At worst, how intense was your neck pain today, on an NRS from 0 to 10, where 0 is no pain and 10 is unbearable pain?
3. How many hours did you have neck pain during the past 24 hours?

After the last intervention, all participants will complete a short satisfaction questionnaire.

Sick leave due to neck pain or other reasons will be monitored throughout the study period.

Participants that receive external, non-trial treatment for their neck pain during the 2-week intervention period, such as osteopathy, physiotherapy, chiropractic manipulation and/or other health care treatment of musculoskeletal complaints, become pregnant, or take NSAIDs and/or other pain medications, will be excluded from the study and classified as dropouts.

Participants will be asked at 1-, 4-, 8-, 12- and 24-weeks, whether they have consulted health professionals or taken supplementary pain medicine, due to their neck complaints.

Adverse events (AEs) will be recorded after each intervention in all four groups, in accordance with the Norwegian Medicines Agency, the European Medicines Agency, and the CONSORT recommendations [31, 43, 44]. Daily questionnaires will be emailed during the intervention period to document AEs.

All participants will complete a daily, validated de-blinding questionnaire during the intervention period, while the chiropractor investigators will fill in a similar questionnaire after each of the five treatment sessions, on how well they thought they maintained blinding for the participants in the manual treatment groups [38].

The participants’ group allocation will be concealed from the research group, because of restricted access to the digitally collected data, containing direct or indirect information about group allocation.

### Data management

All ethical and trial approvals will be stored digitally at Akershus University Hospital, and a secure copy will be held in the archive at Department for Interdisciplinary Health Sciences, Institute of Health and Society, the University of Oslo. The chiropractor investigators will file the Informed Consent forms in their local Trial Master Files, in accordance with Good Clinical Practice (GCP) guidelines and General Data Protection Regulations (GDPR).

The trial data will be collected and stored digitally using the Services for Sensitive data (TSD), University of Oslo, a platform for collecting, storing, analysing, and sharing sensitive data, in compliance with the Norwegian regulations and the GDPR.

A secure, personal, digital Bank-ID login solution and the participant’s unique five-digit study ID-number are linked to the completed participant questionnaires. Only the ID-numbers are filed.

Sociodemographic data will be collected, as well as clinical baseline-, intervention period- and follow-up data. The chiropractor investigator will be responsible for collecting and recording data during the intervention period, while the participant will be responsible for replying to the digital questionnaires sent via e-mail link. The PhD student (AAU) will monitor the study according to the GCP guidelines [45].

Follow-up analysis will be performed on endpoints measured at the end of the intervention period (Day 14) and at 1-, 4-, 8-, 12- and 24-weeks follow-up (see Fig 2). In the case of non-response or missing data, a reminder will be sent via email the next day, followed by telephone contact if the reminder fails to yield a response.

The monitor (AAU) and main PhD supervisor (AC) will conduct individual inspections approximately halfway through the trial, to check that these standards are being upheld at the participating clinics.

### Statistical analysis

Data will be analysed, blinded to the participant’s group allocation by a statistician and the research group, using Stata. Baseline demographics and clinical characteristics at the first chiropractic visit will be presented in tables, as means and standard deviations (SD) for continuous variables, and proportions and percentages for categorical variables. Primary and secondary endpoints will be presented using suitable descriptive statistics for each group and for each time point. The comparison of groups will be performed by a linear mixed model with fixed effects for time, as well as group and interaction between the two. Random effects for participants nested within chiropractors will be included.

Data will be analysed using the intention to treat principle. All randomized patients will be part of the analysis. However, some patients may discontinue treatment or start other/additional treatments during the follow-up. Then, the measurements taken up to that point in time will be used in the analysis, and subsequent measurements will be regarded as missing. A linear mixed model will handle this type of missing values.

The primary endpoint will assess the difference in mean pain intensity change from Day 0 to Day 14 on a numeric rating scale (NRS) 0–10, between CSMT and sham CSMT, CSMT and ibuprofen, and CSMT and placebo medicine, while ibuprofen compared to placebo medicine will be a secondary endpoint.

The other secondary endpoints will assess mean pain intensity and mean duration at different time points, as well as adverse events, blinding success, and treatment satisfaction (see Table 3 for details). Unblinding will occur at the end of statistical analysis when data collection is completed and analysed.

**Table 3:** Primary and secondary endpoints. Chiropractic spinal manipulative therapy (CSMT).

PRIMARY ENDPOINT DESCRIPTION	MEASUREMENT TIMEPOINT	ANALYSIS
Mean pain intensity change (Numerical rating scale 0–10)	From Day 0 to Day 14	Comparison between: CSMT and sham CSMT groups CSMT and ibuprofen groups CSMT and placebo medicine groups
SECONDARY ENDPOINT DESCRIPTION	MEASUREMENT TIMEPOINT	
1. Mean pain intensity change (Numerical rating scale 0–10)	From Day 0 to Day 14	Comparison between: ibuprofen and placebo medicine groups
2. Mean pain intensity change (Numerical rating scale 0–10)	From Day 0 to Days 2–13 in the treatment period From Day 0 to Day 7, 28, 56, 84, 168 post-treatment	Comparison between: CSMT and sham CSMT groups CSMT and ibuprofen groups CSMT and placebo medicine groups ibuprofen and placebo medicine groups
3. Mean duration (hours) of neck pain change	From Day 0 to Days 2–13 and 14 From Day 0 to Day 7, 28, 56, 84, 168 post-treatment	
4. Proportions of participants with mean daily pain intensity reduction of ≤ 50%, ≤ 75%, and 100%		
5. Proportions of participants with mean duration (hours) reduction of ≤ 50%, ≤ 75%, and 100%		
6. Mean number of days with neck pain per week	From the treatment period (14 days) to the periods: Days 2–7, 22–28, 50–56, 78–84 and 162–168 post-treatment	
7. Proportions of participants with mean reduction of number of days with neck pain per week of ≤ 50%, ≤ 75% and 100%		
8. Improvement in Research and development 12 (RAND-12) score	From Day 0 to Day 14, Day 84 and 168 post-treatment	
9. Improvement in Neck Disability Index score		
SECONDARY ENDPOINT DESCRIPTION	MEASUREMENT TIMEPOINT	ANALYSIS
10. Adverse event (AE) analysis	Days 2–13	CSMT, sham CSMT, ibuprofen, and placebo medicineComparison between: CSMT and sham CSMT groups CSMT and ibuprofen groups CSMT and placebo medicine groups ibuprofen and placebo medicine groups
11. Patients’ blinding on numerical rating scale (NRS) 0–10 in relation to receiving real chiropractic spinal manipulative therapy (CSMT) (0 = absolutely unsure and 10 = absolutely sure that real CSMT was received), irrespective of whether the patient received real CSMT or sham chiropractic spinal manipulative therapy (sham CSMT)	Day 0, Days 2–13	Analysis of CSMT and sham CSMT groups Comparison between CSMT and sham CSMT groups
12. Patients’ blinding on numerical rating scale (NRS) 0–10 in relation to receiving ibuprofen (0 = absolutely unsure and 10 = absolutely sure that ibuprofen was received), irrespective of whether the patient received ibuprofen or placebo medicine	Day 0, Days 2–13	Analysis of ibuprofen and placebo medicine groups Comparison between ibuprofen and placebo groups
13. Patients’ and chiropractors’ expectation of treatment efficacy on numerical rating scale (NRS) 0–10 (0 = no expectation of treatment efficacy and 10 = the highest possible expectation of treatment efficacy)	Day 0	Analysis of patients’ and chiropractors’ expectation of treatment efficacy Comparison of patients’ and chiropractors’ expectation of treatment efficacy
14. Patients’ satisfaction of treatment efficacy on a numerical rating scale (NRS) 0–10 (0 = no satisfaction at all, and 10 = highest possible treatment satisfaction)	Day 14	Analysis of patients’ satisfaction of treatment efficacy
ADDITIONAL SECONDARY ENDPOINTS	MEASUREMENT TIMEPOINT	ANALYSIS
15. Sick leave. Number of days and grade of sick leave	Day 0, Week 12, Week 24	Analysis of mean number of days, Day 0 compared to Week 12 and Week 24 post-treatment
16. Validation of user ID-number	All digital questionnaires from Day 0 to study completion, that is to 24 weeks follow-up	Number and proportions of incorrect typing of ID-numbers during digital questionnaire completion
17. Facilitatory/inhibitory factors/dilemmas affecting recruitment. Qualitative focus group interviews with all investigators	April-December 2023	Thematic analysis will be adopted to analyze the interview material

### Statistical power

A power calculation has been performed for the primary endpoint; the mean pain intensity, measured using the NRS. We expect the average NRS score of five at Day 0, with standard deviation (SD), equal to 1 in all groups. This SD is selected since a reduction of one point on the NRS represented a minimal clinically important difference for a given patient, according to Boonstra and colleagues [46]. We will be considering acute neck pain participants with a minimum intensity of 4 on a NRS representing moderate or severe neck pain intensity, i.e., correspond to 4-6/10 and ≥7/10, respectively [46]. A reduction in pain intensity of 60% from Day 0 to Day 14 after baseline is expected in the CSMT group, a 40% reduction is expected in the sham CSMT and ibuprofen groups, while a reduction of 20% is expected in the placebo medicine group. We assume the SD to be 1 at Day 14 after baseline. Due to three comparisons; CMST vs. sham CSMT, CSMT vs. ibuprofen, and CSMT vs. placebo medicine, i.e., multiple testing, the statistical significance level is reduced from 0.05 to 0.017. The sample size necessary to show a statistically significant difference for the primary endpoint with a power of 80%, was estimated to be 43 patients in the CSMT, sham CSMT and ibuprofen groups, and 12, in the placebo medicine group. Since we are going to conduct a multicentre, practice-based study expecting multiple chiropractor investigators to recruit patients, an intra-chiropractor correlation, or cluster effect, is likely to be present in our data. We assume such a cluster effect to be about 25%, a rather conservative estimate. After adjustment for cluster effect, 56 patients will have to be included in the CSMT, sham CSMT and ibuprofen groups. To maintain blinding throughout the statistical analyses, 56 patients will be included in the placebo medicine group. Some dropouts are to be expected. To maintain as high power as possible, we aim to include 80 patients per group, i.e., on average 16 patients per chiropractor investigator, partitioned into four groups with four patients randomly assigned to each group. Thus, a mean difference of 1 on the NRS can be detected, which is the minimal clinically relevant difference [47].

### Ethical considerations

The study is approved by the Norwegian Regional Committee for Medical Research Ethics (REK) (2020/28498); the Data Protection Officer, the Norwegian Social Science Data Services at Akershus University Hospital, Lørenskog, Norway; and by the Norwegian Medicines Agency (EUDRA CT NR. 2021-005483-21). The Declaration of Helsinki will be followed. The clinical trial will adhere to the International Conference on Harmonization-Good Clinical Practice (ICH-GCP) [45]. Participation in the study is voluntary, and participant must give oral and written Informed Consent.

Insurance will be provided by the Norwegian drug liability association (Legemiddelansvarsforeningen) and through The Norwegian System of Patient Injury Compensation (Norsk Pasientskadeerstatning—NPE).

All data in the database will be de-identified using ID-numbers. However, the participants and the chiropractor investigators will know the connection between the ID-number and identity. The final data set will be available to the research group, i.e., PhD student (AAU), PhD supervisors (AC, NKV and MBR) and sponsor (MBR). All data will be stored securely for five years, i.e., digitally at TSD and paper data in a locked cabinet at the Institute of Health and Society (HELSAM), University of Oslo, Norway.

### Safety

The Norwegian Medicines Agency requires that participants experiencing serious AEs or suspected unexpected serious adverse reactions (SUSARs) due to any of the study treatments will be withdrawn and referred to their general practitioner (GP) or hospital emergency department, depending on nature of the event. SUSARs will be reported to the Norwegian Medicines Agency using the CIOMS reporting form. This procedure also adheres to the recommendations from the CONSORT extension for Better Reporting of Harms [31, 48].

The monitor (AAU), whose contact details have been provided, will have the necessary documents, to unblind the medicine that the participant has received.

Should the participant experience a serious AE or SUSAR due to manual treatment, this should then be reported back to the chiropractor investigator, who will guide the participant for appropriate medical assistance and inform the monitor (AAU).

Participant details will be recorded and kept in the local Trial Master Files, and medicines are stored in a secure location.

## Discussion

### Methodological considerations

Methodological shortcomings of previous manual therapy trials have been addressed in several papers [11, 12, 49–52]. Three major shortcomings stand out, namely lack of blinding, intervention modality and insufficient sample size. It is widely acknowledged that manual therapy clinical trials cannot reach the gold standard adopted in pharmacological clinical trials, because the manual therapist cannot be blinded [12], meaning that manual therapy studies can only be single-blinded, and not double-blinded. Unfortunately, very few manual therapy studies have attempted to apply blinding [12].

To date, manual therapy RCTs on acute neck pain have been pragmatic in nature, comparing active treatment arms. Yet arguably, to yield a true net effect, RCTs should aim to utilize a comparison placebo or control group, as well as one or more active treatment group(s). The use of a placebo arm is crucial to extricate the specific effect of the experimental treatment from the non-specific effects of a particular treatment, i.e., placebo response [51, 53–55].

A unimodal approach, as opposed to a multimodal approach, will better isolate the individual effects of SMT. This is because multimodal programs are unable to isolate the impact of each of the single specific interventions. It also introduces a major risk of contextual biases. Considering the severe methodological shortcomings of current manual therapy trials investigating the efficacy of SMT, there is a critical need for higher quality in manual therapy clinical RCTs on acute neck pain and other musculoskeletal pain disorders.

### Limitations of the study design

This prospective clinical multicentre 4-arm placebo RCT will compare the efficacy of manual therapy and pharmacological treatments, for acute neck pain. The trial’s design adheres to recommendations for pharmacological trials as reasonably as possible and is ideal for investigating research questions on treatment efficacy and safety.

However, the use of sham chiropractic manipulation may be influenced by the treatment provider [49]. As a recent methodological review on manual therapy trials pointed out [51], training in performing sham techniques is essential. Therefore, we conducted a workshop to ensure standardization of the treatment protocol. We have also considered that sham procedures should be tailored to the technique they imitate [38, 51, 52]. We consider the use of sham in this trial to be appropriate and as such, aspire to minimizing common RCT methodological shortcomings and improving research quality. The applied sham CSMT intervention has been validated in over 40 clinical studies since its publication in 2015 [38]. We have chosen to adopt this methodology instead of a control group, with the intent of yielding a true net effect of the active intervention and quantifying the placebo effect [39]. This is further strengthened by the incorporation of a placebo medicine group.

We anticipate that the study will have good external validity since it will include multiple chiropractor investigators located across Norway. The disadvantage is possible inter-investigator variability, which we have accounted for in our power calculation, i.e., correction for cluster effect in the upcoming data analyses. We hope that the use of a standardized sham CSMT protocol and participation in a workshop prior to study recruitment will offset this issue somewhat.

A rigorously concealed randomization procedure, use of recommended reliable outcome measures, and patient-reported outcome measures will be utilised, as recommended by a recent methodological review [49].

The main limitation is the risk for recruitment challenges and dropouts, due to an array of reasons. We are including a reasonably large number of chiropractor investigators and are open to recruiting several more, to offset challenges with participant recruitment. Such issues might for example be that participants seek other manual treatments elsewhere or take other pain medication, during the intervention period, which will lead to exclusion from the study. Similarly, participants may not complete the intervention period. For example, participants in the medicine groups may stop taking the medicine before the intervention period is over. Likewise, participants in the manual treatment groups may for unforeseen reasons, not manage to attend all five-treatment sessions during the allotted treatment period. Such cases will be included in the study if the series of questionnaires is correctly completed.

Another possible reason for recruitment challenges might be that participants expect to receive chiropractic treatment when consulting a chiropractic clinic, yet they may be randomized to one of the medicine groups.

The chiropractor investigators cannot be blinded to the manual treatment allocation. However, treatment credibility can be controlled for through de-blinding procedures [52], despite the vast majority of manual therapy trials failing to adopt this approach [51]. The inclusion of a de-blinding questionnaire for both participant and chiropractor investigator in this study should therefore constitute a strength.

It will not be possible to differentiate between the specific effects of treatment and the non-specific (contextual) effects, for example participant-chiropractor interactions. However, we will generate knowledge based on our expectation and satisfaction questionnaires.

A sufficient sample size in a trial has been reported as a commonly, self-acknowledged limitation among manual therapy studies [49]. Underpowered studies can often arise owing to limited recruitment possibilities, because of data collection in private clinics/outpatient centres. We hope to offset this possible issue by recruiting an additional number of chiropractor investigators approximately three months after initial recruitment start depending on the participant recruitment rate. If the rate of recruitment does not fulfil our expectations after the first 6 months of the trial, then appropriate steps will be carried out to rectify this.

### Dissemination

This study is to be completed within three years after its inception. We plan to disseminate the study’s results in peer-reviewed, international scientific journals, in congruence with the CONSORT 2010 Statement. Results will be published regardless of whether they are positive, negative, or inconclusive. The study results will be presented at national and/or international conferences by means of poster(s) and/or oral presentations. A written lay summary will be provided to study participants upon request and disseminated in Norwegian news media.

### User involvement

The recruiting chiropractor investigators will have regular meetings with the research group. They will be users and thus be involved in propagating knowledge generated from this study. The project will also meet with users from Ryggforeningen i Norge, Norway, who will function as discussion partners. When the results of the study are available, they will assist in disseminating the results to patients, patient organizations and healthcare professionals who treat this patient group. This will be conducted through articles in high impact scientific journals, lectures, member magazines and other relevant publications. Ryggforeningen i Norge may also be a driving force to propagate new knowledge from the project quickly.

### Scientific and innovative value

The main aim of the present RCT is to investigate whether CSMT is more efficacious than sham CSMT, ibuprofen and placebo medication in the treatment of acute neck pain.

The lack of trials guiding effective treatment options for clinical decision-making and the paucity of high-quality trials in the field of acute neck pain research is evident. We hope that this trial will provide evidence-based data to the treatment decision-making process. We further hope that the identification of the “right” treatment choice can have a positive effect at the individual level, and impact positively on the individual and the societal costs of musculoskeletal challenges.

If CSMT proves to be effective, it could support this treatment as a viable non-pharmacological option. This is important because some acute neck pain patients do not experience relief from NSAIDs, whilst others experience considerable side effects from NSAIDs, or possess comorbid diseases that contraindicate the use of NSAIDs. Others wish to avoid medication for different reasons. Similarly, if ibuprofen proves to be effective, it can be used based on evidence-based reasoning rather than its current use today, which is not evidence-based.

The study’s unique design guards against the many known biases in published manual therapy RCTs so far. The incorporation of a validated manual sham placebo arm is a major innovative improvement, lifting manual therapy RCTs to the level of pharmacological RCTs, thus making it possible to evaluate true net effect [38]. Furthermore, the study has the potential to rationalize a triangle of importance for stakeholders between patients’-, health care system- and research benefits.

## Supporting information

S1 Checklist SPIRIT 2013 checklist: Recommended items to address in a clinical trial protocol and related documents*. (PDF)

S1 File Proof of external funding. (PDF)

S2 File Proof of ethics approval. (PDF)

S3 File Copy of the protocol that was approved by the ethics committee. (PDF)

## Abbreviations

AAU: Anna Jane Allen-Unhammer
AC: Aleksander Chaibi
AEs: Adverse events
CIOMS: Council for International Organizations of Medical Sciences
CONSORT: Consolidated Standards of Reporting Trials
CSMT: Chiropractic spinal manipulative therapy
GCP: Good Clinical Practice
GDPR: General Data Protection Regulation
GI: Gastrointestinal
GP: General practitioner
HELSAM: Institute of health and society
ICH-GCP: International Conference on Harmonization-Good Clinical Practice
ID: Identification document
MBR: Michael Bjørn Russell
NDI: Neck disability index
NKV: Nina Køpke Vøllestad
NPE: Norsk Pasient Skadeerstatning (The Norwegian System of Patient Injury Compensation)
NRS: Numerical rating scale
NSAID: Non-steroidal anti-inflammatory drug
NYHA: New York Heart Association
PROMs: patient reported outcome measures
QR: Quick response
RAND: Research and development (corporation)
RCT: Randomized controlled trial
REK: Regionale komiteer for medisinsk og helsefaglig forskningsetikk (Regional Committees for Medical and Health Research Ethics
SD: Standard deviation
SMT: Spinal manipulative therapy
SPIRIT: Standard Protocol Items: Recommendations for Interventional Trials
SUSARs: Suspected unexpected serious adverse reactions
TSD: Services for Sensitive Data
UiO: University of Oslo
USD: United States Dollar
