# Supplementary material for: Chiropractic spinal manipulative therapy for acute neck pain: A 4-arm clinical placebo randomized controlled trial. A prospective study protocol
Source: PLoS One. 2023 Dec 7;18(12):e0295115. doi: 10.1371/journal.pone.0295115 (PMC10703251; doi:10.1371/journal.pone.0295115)
Supplement: S1 File — (PDF) [file pone.0295115.s002.pdf]

The following document contains:

- English translation of the external funding document
- Approval from the Norwegian Spine and Back Pain Association for the translated document
- Original copy of the external funding document (in Norwegian)

Yours sincerely,

Anna Allen-Unhammer

PhD student and corresponding author

Department of Interdisciplinary Health Sciences

Institute of Health and Society

Faculty of Medicine

University of Oslo

PROJECT CONTRACT

Agreement between the Back association in Norway and the department of interdisciplinary health sciences, Institute of health and society, the medical faculty,

Postbox 1073 Blindern – 0316 Oslo

The Back association in Norway, from now on called the responsible applicant organization, has from Stiftelsen DAM been assigned

Kr 755 000

for the project

Manuellterapi for akutte nakkesmerter

For the project year 2020

The project number is **2020/FO296562**

Aleksander Chaibi from now on called **project manager** is hereby awarded

Kr 755 000

for the aforementioned project and project year.

The set amount to the project manager will be paid out by the responsible applicant organization within the responsible applicant organizations ordinary economic- administrative routines, such as is specified below.

The responsible applicant organization has the duty to pay out the above amount to the project managers institution every half year, in the form of two similar sized installments as follows:

- The first installment is paid out on the basis of information in the project description, usually the start/mid March. These funds are paid out by the responsible applicant organization to the project managers institution in reasonable time after the contract is signed by the project managers institution and the responsible applicant organization. This payment assumes moreover that the project manager has forwarded the responsible applicant organization the contract as well as the attached *form for transfer of project funds* in a signed and completed format.

- In reasonable time before the responsible applicant organization pays out the second installment to the project managers institution, usually the start/mid September, it is the duty of the project managers institution to inform the responsible applicant organization about the following:
    - Current and planned progress

- Current and planned use of funds
- Planned reporting
- Planned annual/final accounts

(Project managers institution handles the accounts and revisions, while the project manager handles subjects.)

Any comments from Stiftelsen DAM about the use of the funds, has been conveyed in letter format from the responsible applicant organization to the project managers institution.

The project manager and project managers institution has the duty of using the project number in all written correspondence about the project with the responsible applicant organization.

The project managers contact person at the responsible applicant organization is project coordinator at the Back association in Norway p.t. Thor Einar Holmgard, mobile 900 23 422 or e-mail: *extrayggforeningen.no.*

The project manager has the duty, if nothing has been or is to be arranged in written format with the responsible applicant organization, to conduct the project in accordance with the project description which is the basis for the project application, below the comments from the subject committee in Stiftelsen DAM.

The responsible applicant organization has the duty to be the intermediary for all communication between the project manager, project managers institution, and Stiftelsen DAM. The term communication here means questions and inquiries about the project, including development and submission of reports and accounts such that is specified below.

Questions and inquiries about the project shall exclusively be addressed to the responsible applicant organization, that has the duty to give the project manager and project managers institution feedback within a reasonable amount of time. The responsible applicant organization moreover has the duty to go ahead with any current issues to Stiftelsen DAM within a reasonable amount of time, and inform the project manager and project managers institution about the outcome of such initiatives within a reasonable amount of time.

If the project is interrupted, if the overall goals change, or if unforeseen difficulties arise of a financial and/or administrative nature, including the progress of the project, the project managers institution must alert the responsible applicant organization about this immediately and in writing.

Such circumstances may lead to demands for repayment of project funds. Failure to comply with reporting requirements can also lead to demands for repayment of project funds.

The project managers institution has full responsibility for following up on deadlines set by the responsible applicant organization. The responsible applicant organization has the duty to ensure that all reporting deadlines and other follow-up from Stiftelsen DAM as project financier is complied with.

All reporting and follow-up happens electronically via Damnett.no.

For projects that span over several years electronic progress reports should be given during the project period. Progress reports, together with account reports are a part of the application for continued support for initiated projects spanning several years.

For projects carried out at institutions subject to the control of the National Audit Office, accounts certified by the relevant institution’s leader can be accepted. For such projects the institution has the duty to fulfill all of the report deadlines as well as submit certified project accounts with the specification of salary funds used in the project.

The project manager has the duty in their outgoing information about the project to mention Back association in Norway as the responsible applicant organization and Stiftelsen DAM as the project financier.

For research projects the project manager has the duty to prepare a popular science report on the projects content and results. The report must be forwarded to the responsible applicant organization at the same time as the final accounting and final report.

Drammen:

Place: Lier Date: 20.04.21

____

Responsible applicant organization

Back association in Norway

Eirik Moe

Leader in the Back association in Norway

Project manager institution

Department for interdisciplinary health sciences,

Institute for health and society, the

Medical faculty, UiO

____

Aleksander Chaibi

Project manager

Project manager forwards responsible applicant organization / the responsible applicant organization contact person a signed copy of the contract as well as a completed and signer copy of the attached form.

Attachment: *Form for transfer of project funds.*

SKJEMA FOR OVERFØRING AV PROSJEKTMIDLER

Grant year: 2019

Project year: 2020

Project name: Manual therapy for acute neck pain

Project number: 2020/FO296562

Total grant amount from the project owner to the project manager: Kr 755 000

The funds are paid out every half year, c.f. contract

Receiver:

- **Name:** Institute of health and society, UiO
- **Post address:** Pb 1130 Blindern
- **Post code and postal location:** 0318 Oslo
- **Account number:** 7694 05 11077
- **Other markings (Number, code, name etc.):** 13101000-Roald

I confirm that the above information is correct and in accordance with the project descriptions contents and intentions.

______________________________ Place:………………………………Date: …………..

Project manager

Aleksander Chaibi

______________________________ Place:………………………………Date: …………..

Institution with employer responsibility

Department of inter-disciplinary health sciences,

Institute of health and society,

The medical faculty,

UiO

I confirm that the translation of the document Proof of external funding is correct.

Drammen January 9, 2023

Regards,
Eli Steen
CEO Norwegian Spine and Back Pain Association
Phone **l +47** 948 02527
E-mail **l** ryggforeningen@ryggforeningen.no

Postadresse: Ryggforeningen i Norge, postboks 9612 Fjellhagen, 3065 Drammen
Telefon: 69 26 87 00, Organisasjonsnr: 977 07 2204

PROSJEKTKONTRAKT

Avtale mellom Ryggforeningen i Norge og Avdeling for tverrfaglig helsevitenskap, Institutt for helse og samfunn, Det medisinske fakultet, Postboks 1073 Blindern - 0316 Oslo

Ryggforeningen i Norge, heretter kalt **ansvarlig søkerorganisasjon**, har av **Stiftelsen DAM** fått tildelt

kr 755 000

til prosjektet

Manuellterapi for akutte nakkesmerter

for prosjektåret 2020.

Prosjektnummeret er **2020/FO296562.**

Aleksander Chaibi heretter kalt **prosjektansvarlig** tildeles med dette

kr 755 000

til ovennevnte prosjekt og prosjektår.

Det oppsatte beløpet til prosjektansvarlig vil bli utbetalt av ansvarlig søkerorganisasjon innenfor ansvarlig søkerorganisasjons ordinære økonomisk-administrative rutiner, slik dette spesifiseres nedenfor.

Ansvarlig søkerorganisasjon plikter å utbetale ovennevnte beløp til prosjektansvarlig institusjon halvårsvis, i form av to likelydende delanvisninger som følger:

- Første delanvisning utbetales på basis av opplysningene i prosjektbeskrivelsen, vanligvis primo/medio mars. Disse midlene utbetales av ansvarlig søkerorganisasjon til prosjektansvarlig institusjon i rimelig tid etter at kontrakten er signert av prosjektansvarlig institusjon og ansvarlig søkerorganisasjon. Denne utbetalingen forutsetter dessuten at prosjektansvarlig har oversendt ansvarlig søkerorganisasjon kontrakten samt vedlagte *Skjema for overføring av prosjektmidler* i signert og ferdig utfylt stand.

- I rimelig tid før ansvarlig søkerorganisasjon utbetaler andre delanvisning til prosjektansvarlig institusjon, vanligvis primo/medio september, plikter prosjektansvarlig institusjon å informere ansvarlig søkerorganisasjon om følgende:
    - Foreløpig og planlagt fremdrift
    - Foreløpig og planlagt bruk av midler
    - Planlagt rapportering
    - Planlagt års/sluttregnskap

(Prosjektansvarlig institusjon håndterer regnskap og revisjon, mens prosjektansvarlig håndterer fag.)

Eventuelle kommentarer fra Stiftelsen DAM om bruken av midlene, er blitt formidlet i brevs form fra ansvarlig søkerorganisasjon til prosjektansvarlig institusjon.

Prosjektansvarlig og prosjektansvarlig institusjon plikter å bruke prosjektnummeret i all skriftlig kommunikasjon om prosjektet med ansvarlig søkerorganisasjon.

Prosjektansvarliges kontaktperson hos ansvarlig søkerorganisasjon er prosjektkoordinator hos Ryggforeningen i Norge p.t. **Thor Einar Holmgard**, mobil 900 23 422 eller e-post: extra@ryggforeningen.no.

Prosjektansvarlig plikter, om ikke annet er blitt eller blir avtalt skriftlig med ansvarlig søkerorganisasjon, å utføre prosjektet i samsvar med prosjektbeskrivelsen som ligger til grunn for prosjektsøknaden, herunder kommentarene fra fagutvalget i Stiftelsen DAM.

Ansvarlig søkerorganisasjon plikter å være mellomledd for all kommunikasjon mellom prosjektansvarlig, prosjektansvarlig institusjon og Stiftelsen DAM. Med uttrykket kommunikasjon forstås her spørsmål og henvendelser om prosjektet, herunder utvikling samt innsending av rapporter og regnskap slik dette spesifiseres nedenfor.

Spørsmål og henvendelser om prosjektet skal utelukkende rettes til ansvarlig søkerorganisasjon, som plikter å gi prosjektansvarlig og prosjektansvarlig institusjon tilbakemelding innen rimelig tid. Ansvarlig søkerorganisasjon plikter dessuten ved behov å gå videre med aktuelle problemstillinger til Stiftelsen DAM innen rimelig tid, og informere prosjektansvarlig og prosjektansvarlig institusjon om utfallet av slike initiativ innen rimelig tid.

Hvis prosjektet avbrytes, om overordnede mål endres, eller dersom det oppstår uforutsette vansker av økonomisk og/eller administrativ art, herunder fremdriften av prosjektet, skal prosjektansvarlig institusjon varsle ansvarlig søkerorganisasjon om dette umiddelbart og skriftlig.
Slike forhold kan medføre krav om tilbakebetaling av prosjektmidler. Også manglende overholdelse av rapporteringskrav kan medføre krav om tilbakebetaling av prosjektmidler.

Prosjektansvarlig institusjon har det hele og fulle ansvaret for å følge opp tidsfrister som fastsettes av ansvarlig søkerorganisasjon. Ansvarlig søkerorganisasjon plikter å påse at alle

rapporteringsfrister og annen oppfølging ovenfor Stiftelsen DAM som prosjektfinansiør blir overholdt.

All rapportering og oppfølging skjer elektronisk via Damnett.no.

Ved flerårige prosjekter skal det avgis elektronisk framdriftsrapport i prosjektperioden. Framdriftsrapporten, sammen med regnskapsrapporteringen er en del av søknaden om videre støtte for igangsatte flerårige prosjekter.

Ved prosjekter gjennomført ved institusjoner underlagt Riksrevisjonens kontroll, kan regnskap attestert av den aktuelle institusjonens leder aksepteres. Ved slike prosjekter forplikter institusjonen seg til å innfri alle rapporteringsfrister samt avgi attestert prosjektregnskap med spesifikasjon av lønnsmidler brukt i prosjektet.

Prosjektansvarlig plikter i sin utadrettede informasjon om prosjektet å nevne Ryggforeningen i Norge som ansvarlig søkerorganisasjon og Stiftelsen DAM som prosjektfinansiør.

Ved forskningsprosjekter plikter prosjektansvarlig å utarbeide en populærvitenskapelig rapport om prosjektets innhold og resultater. Rapporten skal oversendes ansvarlig søkerorganisasjon samtidig med sluttregnskap og sluttrapport.

Drammen, 21.04.21

Ansvarlig søkerorganisasjon

Ryggforeningen i Norge
Eirik Moe

Leder i Ryggforeningen i Norge

Sted: Lier Dato: 20.4.21

Prosjektansvarlig Institusjon

Avdeling for tverrfaglig helsevitenskap, Institutt for helse og samfunn, Det medisinske fakultet, UiO.

Aleksander Chaibi

Prosjektansvarlig

Prosjektansvarlig oversender ansvarlig søkerorganisasjon v/ ansvarlig søkerorganisasjon kontaktperson ett signert eksemplar av kontrakten samt ett ferdig utfylt og signert eksemplar av vedlagte skjema.

Vedlegg: *Skjema for overføring av prosjektmidler.*

SKJEMA FOR OVERFØRING AV PROSJEKTMIDLER

Bevilgningsår: 2019

Prosjektår: 2020

Prosjektnavn: Manuellterapi for akutte nakkesmerter

Prosjektnummer: 2020/FO296562

Samlet bevilgningsbeløp fra prosjekteier til prosjektansvarlig: Kr 755 000
Midlene utbetales halvårsvis, jf. kontrakt.

Mottaker:

- **Navn:** Institutt for helse og samfunn, UiO
- **Postadresse:** Pb 1130 Blindern
- **Postnummer og poststed:** 0318 Oslo
- **Kontonummer:** 7694.05.11077
- **Eventuell merking (nr., kode, navn etc.):** 13101000-Roald

Jeg bekrefter at ovenstående opplysninger er riktige og i samsvar med prosjektbeskrivelsens innhold og intensjoner.

Prosjektansvarlig
Aleksander Chaibi

Sted: Oslo Dato: 19/4-21

Institusjon med arbeidsgiveransvar
Avdeling for tverrfaglig helsevitenskap, Institutt for helse og samfunn, Det medisinske fakultet, UiO

Sted: Lier Dato: 20.4.21
