# Supplementary material for: Chiropractic spinal manipulative therapy for acute neck pain: A 4-arm clinical placebo randomized controlled trial. A prospective study protocol
Source: PLoS One. 2023 Dec 7;18(12):e0295115. doi: 10.1371/journal.pone.0295115 (PMC10703251; doi:10.1371/journal.pone.0295115)
Supplement: S2 File — (PDF) [file pone.0295115.s003.pdf]

The following document contains:

- English translation of the ethics approval document
- Approval from the Regional Committee for Medical and Health Research Ethics for the translated document
- Original copy of the ethics approval document (in Norwegian)

Yours sincerely,

Anna Allen-Unhammer

PhD student and corresponding author

Department of Interdisciplinary Health Sciences

Institute of Health and Society

Faculty of Medicine

University of Oslo

REK

Michael Bjørn Russell

28498 Chiropractic treatment of acute neck pain

Research institute responsible: Akershus University Hospital HF

Applicant: Michael Bjørn Russell

REKs evaluation

We refer to the application regarding project changes received 09.06.2020 for the aforementioned research project (previous REK-ref: 2019/1237, EudraCT nr. 2018-004602-26). The application is processed by the leader of REK-south-east with the power of attorney, and authority from the Health Research Act § 11.

The changes imply:

- New start date is set to 01.08.2020, and new end date is set to 31.07.2023.

- It is requested that the design is changed from 5 to 4 arms. From protocol: "1) Chiropractic spinal manipulative therapy (CSMT), 2) CSMT sham manipulation (placebo), 3) Ibuprofen medication (ibuprofen 600mg), and 4) Placebo medication".

- Updated protocol is attached, as well as 3 questionnaires.

- The information sheet is updated according to the changes, as well as according to the committees terms for approval in the letter dated 22.11.2019.

The committees leader has evaluated the application and has no research ethical objections to the change of the project.

The committee assumes that the other conditions that were given in the letter dated 22.11.2019 are fulfilled.

-The study cannot be initiated before a response from the Norwegian Medicines Agency is available.

-The response from the Norwegian Medicines Agency must be forwarded to the committee. (The form “Change and/or inquiry” can be used for this)

Decision

Approved

REK has taken a research ethics evaluation of the changes in the project, and approves the project as it is now, cf. the health research act § 11.

We wish to make you aware that according to the new personal data act there must also be a basis for treatment according to the personal protection regulation. It must be anchored to its own institution.

The permission is granted under the condition that the project is performed such as it is described in the application, application for changes, updated protocol and the decisions which follow from the health research act with regulations.

With regards

ing

Recruiting kiros | To Manager
Team Email | Done
Reply & Delete | Create New

Quick Steps

Move | Rules | OneNote

Move

Unread/ Read | Categorize | Follow Up

Tags

Search People
Address Book
Filter Email

Find

Reply | Reply All | Forward

ons. 04.01.2023 13:47

Anne Cathrine Bjercke

RE: 28498 Kiropraktikk behandling av akutte nakkesmerter

To Anna Jane Allen-Unhammer

You replied to this message on 04.01.2023 14:16.
This message is part of a tracked conversation. Click here to find all related messages or to open the original flagged message.

Hei igjen

Her er en engelsk oversettelse av svaret:

To the project manager

We refer to the enclosed english version of the decision made by REC comittee D 29.06.2020, and translated by Anna Allen-Unhammer

We take note of the translated decision, and we do not object to it being published.

Best regards
Anne Cathrine Bjercke
Executive
REC South-East D

Region:	Saksbehandler:	Telefon:	Vår dato:	Vår referanse:
REK sør-øst D	Silje U. Lauvrak	22845520	29.06.2020	28498
			Deres referanse:	

Michael Bjørn Russell

28498 Kiropraktikk behandling av akutte nakkesmerter

Forskningsansvarlig: Akershus universitetssykehus HF

Søker: Michael Bjørn Russell

REKs vurdering

Vi viser til søknad om prosjektendring mottatt 09.06.2020 for ovennevnte forskningsprosjekt (tidligere REK-ref.: 2019/1237, EudraCT nr. 2018-004602-26). Søknaden er behandlet av leder for REK sør-øst på fullmakt, med hjemmel i helseforskningsloven § 11.

Endringene innebærer:

- Ny startdato er satt til 01.08.2020, og ny sluttdato er satt til 31.07.2023.

- Det søkes om å endre designet fra 5 til 4 armer. Fra protokoll: "1) Chiropractic spinal manipulative therapy (CSMT), 2) CSMT sham manipulation (placebo), 3) Ibuprofen medication (ibuprofen 600 mg), and 4) Placebo medication".

- Oppdatert protokoll er vedlagt, samt 3 spørreskjemaer.

- Informasjonsskrivet er eoppdatert i henhold til endringene, samt i henhold til komiteens vilkår for godkjenning i brev datert 22.11.2019.

Komiteens leder har vurdert søknaden og har ingen forskningsetiske innvendinger mot endringen av prosjektet.

Komiteen forutsetter at de øvrige vilkårene som ble gitt i brev datert 22.11.2019 oppfylles:

- Studien kan ikke igangsettes før svar fra SLV foreligger.

- Svaret fra SLV skal ettersendes komiteen. (skjema 'Endring og/eller henvendelse' kan benyttes til dette)

Vedtak

Godkjent

REK har gjort en forskningsetisk vurdering av endringene i prosjektet, og godkjenner prosjektet slik det nå foreligger, jf. helseforskningsloven § 11.

Vi gjør samtidig oppmerksom på at etter ny personopplysningslov må det også foreligge et behandlingsgrunnlag etter personvernforordningen. Det må forankres i egen institusjon.

Tillatelsen er gitt under forutsetning av at prosjektet gjennomføres slik det er beskrevet i søknaden, endringssøknad, oppdatert protokoll og de bestemmelser som følger av helseforskningsloven med forskrifter.

Med vennlig hilsen

Finn Wisløff
Professor em. dr. med.
Leder

Silje U. Lauvrak
Seniorrådgiver

Kopi til: Akershus universitetssykehus HF

Klageadgang
Du kan klage på komiteens vedtak, jf. forvaltningsloven § 28 flg. Klagen sendes til REK sør-øst D. Klagefristen er tre uker fra du mottar dette brevet. Dersom vedtaket opprettholdes av REK sør-øst D, sendes klagen videre til Den nasjonale forskningsetiske komité for medisin og helsefag (NEM) for endelig vurdering.
