# Supplementary material for: Chiropractic spinal manipulative therapy for acute neck pain: A 4-arm clinical placebo randomized controlled trial. A prospective study protocol
Source: PLoS One. 2023 Dec 7;18(12):e0295115. doi: 10.1371/journal.pone.0295115 (PMC10703251; doi:10.1371/journal.pone.0295115)
Supplement: S3 File — (PDF) [file pone.0295115.s004.pdf]

Clinical study

Chiropractic spinal manipulative therapy for acute neck pain: a 4-armed clinical placebo randomized controlled trial

Eudra CT number: 2021-005483-21

Protokollkode 15-2021

Sponsor
Akershus University Hospital
Michael Bjørn Russell
Sykehusveien 27
1478 Lørenskog, Oslo
Norway

Staten legemiddelverk ref. 21-28147-7

List of the participating chiropractors

**NAVN**	**KLINIKK**
**Ole Sommerseth**	**Atlasklinikken (Oslo)**
**Øivind Kolstad**	**Eureka (Bodø) (nordland)**
**Kyrre Neverdal**	**Eureka (Bodø) (Nordland)**
**Maja Bjørnli**	**Helse i Sentrum (Oslo)**
**Are Nikolai Skjøthaug**	**Ganddal terapi og trening (Sandnes, Rogaland)**
**Hege Herstad**	**Grini kiropraktikk og helse (Røa, Viken)**
**Gro Røsok**	**Kiropraktorhuset Elverum (Innlandet)**
**Kari Ødegaard Hommedal**	**Kiropraktor i Levanger (Trondelag)**
**Siri Meckelborg**	**Kolbotn medisinskesenter**
**Martin Pran**	**KFA (Storo) (Oslo)**
**Lovisa Klingenberg**	**Fokusklinikken, Økern (Østre Aker vei 19, 0581 Oslo)**
**Nina Søndenå**	**Raftklinikken.no, (Rogaland)**
**Marie Hermansen**	**Råholt Kiropraktorsenter (Viken)**
**Thomas Jakobsen**	**Sarpsborg Kiropraktorklinikk (Viken)**
**Mari Grønlund Kviteberg**	**Tromsø kiropraktikk og helse (Troms og Finnmark)**
**Øyvind Nilsen**	**Tønsberg Kiropraktorklinikk (Vestfold/Telemark)**
**Dag Christian Andersen**	**Ullensaker Kiropraktorsenter (Viken)**

Contents

Eudra CT number	1
Protokoll kode	1
Sponsor information	1
Statens legemiddelverk ref. number	1
List of chiropractors	2
Contents	3-4
Abbreviation	6
Background	7
Introduction	7
Neck pain is a frequent and episodic condition	7
Spinal manipulation is one method to treat neck pain	7
Evidence for effect of spinal mainipulation for neck pain	8
Metholological issues	8
Scientific rationale for the project	8
Patient, health care system and research benefits and risk	8
Patients’ benefits	8
Health care system benefits	8
Research benefits	9
Patients’ risk	9
Benefit-risk ratio	9
Hypothesis, aims and study approach	9
Hypothesis	9
Short term aims	9
Long term aims	9
Study approach	9
Methodology	9

Design and method selection	9
Study design	10
Randomization	10
Inclusion criteria	10
Exclusion criteria	10
Expected participation flow diagram	10
Setting	12
Clincal trial	12
Interventions	12
Ethical considerations	12
Dissemination	13
Analyses	13
Data recording	13
Schedule of activities/events	13
Baseline	13
Treatment period	14
Posttreatment	14
Objectives	14
Primary objectives	14
Secondary objectives	14
Primary end-point	15
Secondary end-point	15
Blinding	15
Adverse events	16
Serious adverse events and suspected unexpected serious adverse event	16
Expectation	16
Satisfaction	16
Statistical power	16

Participants, organization and collaboration	16
Registration and publication of trial protocol	16
Budget	16
Progression plan	17
Publication plan	17
Plan for activities, visibility and dissemination	17
Plan for implementation and visibility	17
User involvement	17
Innovative and scientific value	17
Limitations and strengths	18
Delegation log (Delegeringslogg)	19
References	20

Abbreviation

AE – Adverse event

CIOMS - The Council for International Organizations of Medical Sciences

Class IV - Severe limitations. Experiences symptoms even while at rest. Mostly bedbound patients. Consort - CONsolidated Standards of Reporting Trials CSMT - Chiropractic spinal manipulative therapy

DC – doctor of chiropractic

DrMedSci – doctor of medical science

GCP – Good Clinical Practice

GP(s) – general practitioner(s)

HVLA - high-velocity low amplitude

ICH - International Council for Harmonisation of Technical Requirements for Registration of Pharmaceuticals for Human Use

NRS – numeric rating scale

NYHA - The New York Heart Association

NSAID(s) – non-steroidal anti-inflammatory drug(s) PhD – doctor of philosophy PT - fysiotherapist

RCT(s) - randomized controlled trial(s)

RSI - reference safety information

SAE – serious adverse events

SD – standard deviation

SMT - spinal manipulative therapy

SUSAR – suspected unexpected serious adverse events TSD – services for sensitive data UiO – University of Oslo

US – United States

USD – United States dollars

Chiropractic spinal manipulative therapy for acute neck pain: a 4-armed clinical placebo randomized controlled trial

Background

The Global Burden of Disease study ranks musculoskeletal neck pain as the most common disability worldwide (1). This study will highlight and validate chiropractic manual-therapy for acute neck pain. The applied methodology of the study will aim towards the highest research standards possible for manual-therapy randomized controlled trials (RCTs), thus avoiding typical methodological shortcomings from previous manual-therapy studies, including lack of a placebo comparison group. Our study aims to establish the efficacy of chiropractic manual-therapy in the management of acute neck pain compared to other relevant treatment options, in order to enhance evidence-based clinical practice.

The Global Burden of Disease study states that musculoskeletal disorders, more specifically lower back and neck pain in the period 1990-2015, were the leading cause of non-fatal disability in almost all age groups (2, 3). The point prevalence estimate of neck pain was 4.9-7.6% (4, 5). In other words, >400 000 Norwegians were suffering from neck pain on any given day per 3rd quarter in 2021. The total cost to society is unknown; however, a recent review estimated that the annual spending on personal health care and public health for low back and neck pain combined was USD 87.6 billion in the US alone (6). The World Health Organization recently acknowledged that musculoskeletal health conditions contribute greatly to disability across the life-course in all regions of the world. As much as 46% of sickness absence and 33% of disability pensions are directly related to musculoskeletal disorders (7, 8).

This study will investigate four different treatment options
1) Chiropractic spinal manipulative therapy (CSMT)
2) CSMT sham manipulation (placebo)
3) Ibuprofen medication (ibuprofen 600 mg)
4) Placebo medication.

Chiropractors treat musculoskeletal disorders, but the profession is not fully integrated into the Norwegian health care sector, since only about 8% of general practitioners (GPs) refer neck pain patients to manual-therapy (9). In comparison, NSAIDs are the most frequently prescribed medications by GPs worldwide and are widely used for patients with lower back pain (10). However, evidence-based data are missing for acute neck pain patients treated with NSAIDs (5, 11). Our study aims to provide evidence-based data on the four treatments option mentioned above, so that clinicians and patients can make decisions that are more informed. We hope that our study will also facilitate better inclusion of the chiropractic profession into the primary health care team, through acknowledgement of high-quality chiropractic research.

Introduction

Neck pain is a frequent and episodic condition

Episodes of neck pain of varying duration are very common in the general population, as up to 50% of adults experience neck pain within one year, and the recurrence of neck pain is frequent (12, 13).

Spinal manipulation is one method to treat neck pain

Neck pain is the second most common complaint treated by chiropractors, after lower back pain (14, 15). More than 90% of Norwegian chiropractors use various manipulation- and/or mobilization techniques, often combined with soft tissue techniques, exercise modalities, and general advice (16). Spinal manipulative therapy (SMT) is defined as a passive controlled manoeuvre that uses a directional high-velocity low amplitude (HVLA) thrust directed at a specific joint past the physiological range of motion without exceeding the anatomical limit (17). SMT may relieve neck pain by stimulating neural inhibitory systems at different spinal cord levels, through activation of

various central descending inhibitory pathways (18-23). However, the physiological mechanisms of pain relief are not fully understood, and contextual factors need to be identified (24).

Evidence for effect of spinal manipulation for neck pain
A Cochrane review included 51 trials (2,920 participants) assessing manual-therapy for neck pain (15). Eighty percent (41/51) of the studies were found to be of very low or low quality. Thus, uncertainty regarding the effectiveness of manual-therapy for neck pain remains.

Methodological issues
The methodological quality of manual-therapy RCTs for a range of musculoskeletal disorders is frequently criticised for being too low. Furthermore, the lack of blinding is regarded as a major shortcoming. Manual-therapy RCTs cannot be double-blinded as in pharmacological RCTs, since the practitioner providing the interventions in a manual-therapy study cannot be blinded. It is generally recommended that the placebo intervention should resemble the active treatment in terms of the procedure, treatment frequency and the time spent with the patient, to allow for similar expectations in both groups (25). However, an appropriate placebo for manual-therapy still lacks consensus amongst clinical and academic experts (26). For this reason, previous manual-therapy studies were pragmatic and/or used “no treatment” as the control group. Our research group invented and validated CSMT sham intervention in two RCTs (27, 28). The results indicated that it was possible to conduct a chiropractic sham-controlled manual-therapy RCT, where the study participants were blinded throughout the study period, with 12 interventions over 3 months. The manual placebo method has since been used with success in >30 international clinical trials (29-33). Thus, manual-therapy RCTs can be single-blinded.

Scientific rationale for the proposed study
Acute neck is very common in the general population and often cause disability of shorter or longer time periods. Unfortunately, the efficacy of CSMT and the efficacy of NSAIDs on acute neck pain is unknown (15). This 4-armed RCT will likely provide evidence for the efficacy of CSMT as well as NSAIDs.
Our study will compare CSMT to a credible and validated CSMT sham manipulation. Two pharmacological arms are also to be introduced, in the form of an ibuprofen medication and a placebo medication treatment arm. The 4-armed placebo-controlled RCT will assess the efficacy in the following four treatment groups:
1) CSMT
2) CSMT sham manipulation (placebo)
3) Ibuprofen 600 mg
4) Placebo medication.

Patient, health care system and research benefits and risk
Should our study find that chiropractic manual-therapy and medication treatments are effective, then our study will provide evidence-based data for non-pharmacologic and pharmacological treatment.

<u>Patients’ benefits</u>

- Those who prefer chiropractic manual-therapy
- Those who do not respond-, prefer to avoid-, and/or do not tolerate ibuprofen
- Those who do not respond to physiotherapy and/or other manual treatment
- Relieve the burden of pain, speed up recovery, reduce sick leave

<u>Health care system benefits</u>

- Reduce costs of inert treatments, reduce sick leave, reduce referrals to 2nd and 3rd line health care, reduce referrals to image diagnostics, and filling knowledge gaps among chiropractors, other manual therapists and physicians.

<u>Research benefits</u>

- The methodology of blinding in a manual-therapy RCT by including a valid CSMT sham manipulation arm is of importance to establish efficacy with certainty. Additionally, it can be applied in future manual-therapy RCTs.

<u>Patients' risk</u>

- Real and sham CSMT side effect are muscle soreness
- Ibuprofen side effects, i.e. nausea, abdominal pain, dyspepsia, allergic reactions, headache and skin rash.

<u>Benefit-risk ratio</u>

The overall benefit-risk ratio is in favour of CSMT and ibuprofen treatment, as the probability for treatment effect by far exceeds the very low risk for serious adverse events.

Hypothesis, aims and study approach

Hypothesis

CSMT is effective in the management of acute neck pain, more so than CSMT sham manipulation, Ibuprofen medication, and placebo medication.

Aims

<u>Short term aims</u>

Relief of neck pain. Inspire towards higher scientific methodological quality of manual-therapy RCTs by specifically including a placebo intervention arm.

<u>Long term aims</u>

Relief of neck pain. Provide evidence-based data for non-pharmacological and pharmacological treatment.

Study approach

a) Design a 4-armed multi-center practice-based placebo-controlled RCT on the effect of chiropractic manual-therapy for acute neck pain
b) Conduct the RCT
c) Publish results in high impact scientific journals
d) Provide evidence-based results for health care providers
e) Present results to the public including national and international media
f) Present results to professionals at national and international meetings and congresses

Methodology

The project group has finalised the study protocol and will obtain the necessary approvals before initiation, see the ethical consideration section below for details. The PostDoc has recruited chiropractors for the project and will together with the PhD student and the study group thoroughly prepare them for the rigorous methodological approach through a workshop. The PhD student will monitor the clinical trial throughout the data collection period and ensure participants and interventional therapist protocol compliance. Data analysis will consist of a collaboration between the PhD student, project leader, supervisors, PostDoc and statistician, with the latter as a key figure in power calculation and the data analysis. The PhD student will draft publications. For specific time points, see progression and activity plan.

<u>Design and method selection</u>

The 4-armed placebo-controlled RCT will assess:

1) CSMT
2) CSMT sham manipulation
3) Ibuprofen medication
4) Placebo medication.

Our research design selection was based on our previous experience that it is possible to conduct chiropractic placebo-controlled RCTs (34). It will not possible to blind the manual therapist, i.e., the chiropractor. However, the two pharmacological arms will be double-blinded. The study participants, the monitor (PhD student) and the statistician are all to be blinded.

Study design

A total of 17 chiropractors from larger Norwegian cities have been recruited (page 3) and will be expanded with up to 8 chiropractors, distributed by gender and geography, equalling 1.6-3.0% of all chiropractors registered in Norway in 2016, according to the Norwegian Chiropractic Association. Each chiropractor is assigned to a chiropractor ID number used in the TSD database and when analysing data. The chiropractor know his/hers own ID number and the study group also know the ID number for each chiropractor. The ID numbers is omitted from page 3, the chiropractor list, as others can retrieve this document. Thus, it is not possible to identify personal data from the participating chiropractors in the TSD database, i.e. deidentified (avidentifisert). The study participants presenting to each chiropractor will be block-randomized into one of four study groups based on a computer-generated algorithm. Each chiropractor will include 16 participants, four into each arm. A total of 320 participants will be enrolled in the RCT, within 12 months (see participant's flow diagram).

Randomization

Each chiropractor will receive four sets each consisting of four concealed envelopes. Each set of four concealed envelopes contain one allocation to each of the four arms. The randomization log has was conducted by a statistician. The consecutive participant will draft a concealed envelop, and then hand it over to the chiropractor, since the participant is not allowed to see if whether it is real or sham chiropractic to be received. If the participant is randomized to medicine, neither the chiropractor nor the participant will know whether the participant receive ibuprofen or placebo, since Kragerø tablet production will package the medicine according to the randomization log provided by the statistician. The participant randomization ID number is filed in the TSD database, while no personal data are filed, i.e. deidentified (avidentifisert). The chiropractors keep a secure list of participant's name, mobilphone and e-mail address and ID numbers under lock, and the PhD student also have this list, as she has to e-mail the participants when they need to fill in the different questionnaires during the study. She will keep this list under lock in the Trial Master File. To ascertain the questionnaires Bank ID are used, but the Bank ID data are not filed. The participants ID number connects questionnaires from each participant.

Inclusion criteria

1. Eligible participants are between the age of 18 and 59 years old
2. Acute non-radicular neck pain, i.e., grade 1 or 2 according to the classification by the Bone and Joint Decade 2000-2010 Task Force on neck pain (35).
3. Onset of the present episode ≤2 weeks prior to the 1st chiropractic visit.
4. Moderate, severe or very severe pain intensity, i.e., ≥4, on a numeric rating scale (NRS) 0-10.
5. Pain free for at least four consecutive weeks prior to the present pain episode.
6. Not treated by a chiropractor during the past 6 months.
7. Participants must accept not to seek other manual and/or pharmacological treatments for their acute neck pain during the intervention period.
8. Non-pregnant women. Women in doubt shall have a negative fertility test before inclusion.

Exclusion criteria

1. Contraindication to ibuprofen
    a. active peptic ulcer
    b. gastrointestinal bleeding
    c. previous repeated episode (≥2 detected events) with peptic ulcer or gastrointestinal bleeding
    d. previous gastrointestinal bleeding or ulcer using NSAIDs
    e. hypersensitivity to ibuprofen
    f. asthma induced by acetylsalicylic acid or other NSAIDs
    g. urticarial

h. rhinitis
i. severe heart failure (NYHA class IV)
j. renal failure (glomerulus infusion <30 ml/min),

2. Ingestion of NSAIDs
3. Prescription opioids within ≤14 days
4. Ingestion of any analgesics within ≤24 hours prior to baseline
5. On prescribed antidepressant.
6. Major psychiatric disorder.
6. Pregnancy or intension to be pregnant
7. Contraindication to SMT.
8. Signs of spinal radiculopathy including progressive neurological deficit
9. Upper cervical spine instability (positive Sharp-Purser test)(36)
10. Previous fracture in the cervical and/or thoracic spine
11. Previous cervical spine surgery,
12. Recent (<6 months) severe physical trauma to the head, neck or thoracic spine within the previous 6 months
13. Concomitant low back pain with moderate, severe or very severe pain intensity (≥4 on a NRS)
14. Current chronic pain (defined as ≥3 months duration)
15. Rheumatoid arthritis
16. Recent (<2 weeks) acute respiratory infection with fever
17. Any presence of ischemic symptoms upon examination
18. Horner's syndrome
19. Medical history of arterial anomalies
20. History of connective tissue disorder
21. Familial history of cervical artery dissection
22. Other vascular disorders (37)
23. Inability to understand instructions given in the Norwegian language
24. Inability to fill out digital questionnaires
25. Other reasons to exclude the patient as deemed necessary by the chiropractor.

Expected participation flow diagram

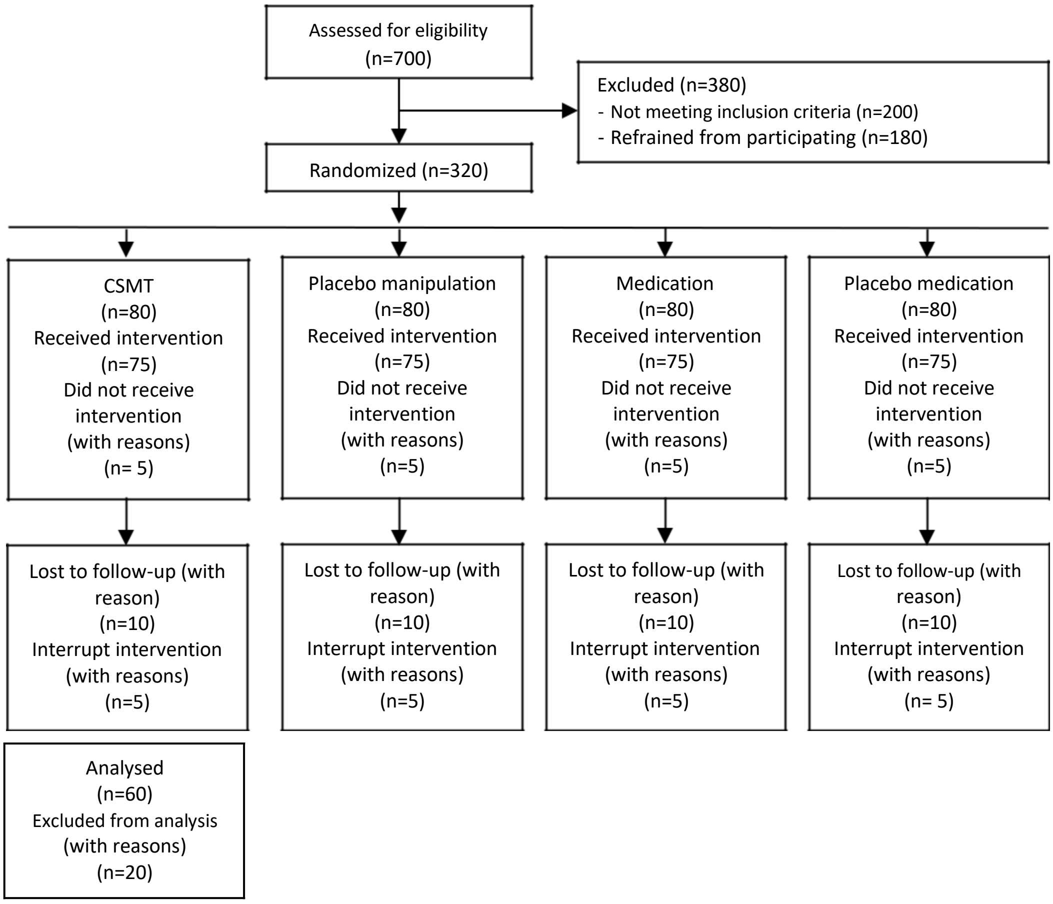

Analysed (n=60)	Analysed (n=60)	Analysed (n=60)
Excluded from analysis (with reasons) (n=20)	Excluded from analysis (with reasons) (n=20)	Excluded from analysis (with reasons) (n=20)

<u>Setting</u>
Division for Research and Innovation, Akershus University Hospital, Lørenskog, Norway and Campus Akershus University Hospital, University of Oslo, Nordbyhagen, Norway. The majority of the project group work at Faculty of Medicine, Institute of Health and Society, University of Oslo, Norway. All interviews, assessments and interventions will be conducted by experienced chiropractors at Norwegian chiropractic clinics. The Ibuprofen and the placebo medication group will receive medication at the chiropractic clinic that recruited the participant.

<u>Clinical trial</u>
The clinical trial will adhere to the protocol, the ICH-GCP and Norwegian regulations for clinical trials. (Clinical Trials are mainly regulated by international and national laws, and the European Directive 2001/20/EC, which is fully implemented in the Norwegian Regulation relating to Clinical Trials on Medicinal Products for Human Use.)
Reference safety information (RSI) is the SmPC for ibuprofen 600 mg.

The clinical trials consist of three stages: Run-in, intervention (treatment) and follow-up. The CSMT, and the CSMT sham manipulation, group 1 and 2 will include five intervention sessions over 10-12 days, i.e., three sessions the 1st week and two sessions the 2nd week unless they recovered or experienced a serious adverse event (AE) during this period. The Ibuprofen and the placebo medication, group 3 and 4 will receive a package with 36 tablets, i.e., 600 mg ibuprofen or placebo to be taken three times per day for 12 days.

<u>Interventions</u>
The first active treatment will consist of CSMT (31), i.e., a specific contact, high-velocity, low-amplitude, spinal thrust manipulation directed to spinal biomechanical dysfunction in the cervical and/or thoracic spinal column, as diagnosed by standard chiropractic tests, in accordance with their clinical judgment. No additional co-interventions or specific advice apart from reassurance of "business as usual" and encourage of normal activities will be given to this group during the trial period, in accordance with practice guidelines.

The CSMT sham intervention group will consist of CSMT sham manipulation, i.e., a broad non-specific contact, low-velocity, low-amplitude sham push manoeuvre in a non-therapeutic directional line (34, 39, 40). All the non-therapeutic contacts will be performed outside of the spinal column with adequate joint slack and without soft tissue pre-tension, so that joint cavitations do not occur. No additional co-interventions or specific advice apart from reassurance of "business as usual" and encourage of normal activities will be given to this group during the trial period.

The Ibuprofen and placebo medication groups will receive 36 tablets ibuprofen 600 mg or 36 tablets placebo (three daily administrations for 12 days) (41, 42). All interventions will be free of charge for the included participants. No additional co-interventions or specific advice apart from reassurance of "business as usual" and encourage of normal activities will be given to this group during the trial period.

Participants in all four groups who do not experience spontaneous improvement, will after study completion (after 6 months follow-up) be offered a free of charge chiropractic assessment and treatment, if necessary, at the location they were first randomized, for up to five treatment sessions. Similarly, participants with a relapse of neck pain during the follow-up period will also be offered a second round of the same intervention, free of charge after 6 months follow-up.

<u>Ethical considerations</u>
The study is approved by the Norwegian Regional Committee for Medical Research Ethics (REK) (REK: 2020/28498). The study has been approved by Personvernombudet, the Norwegian Social Science Data Services at Akershus University Hospital, Lørenskog, Norway, and the Norwegian

Medicines Agency (EUDRA CT NR. 2021-005483-21), prior to initiation of the study. The declaration of Helsinki is followed. All data will be deidentified (avidentifisert) by the use of ID number for both chiropractors and participant. To ascertain the questionnaire Bank ID is used, but the Bank ID data are not filed. The participants ID number connects questionnaires from each participant. Data will be stored electronically at Services for Sensitive Data (TSD), University of Oslo for five years. Insurance is provided through “The Norwegian System of Compensation to Patients” (NPE) and through the Legemiddelverkets ansvarsforsikringen, Norwegian Medicines Agency. A stopping rule was defined for withdrawing participants from this study in accordance with recommendations in the CONSORT extension for Better Reporting of Harms. If a participant reports to their chiropractor or research staff a severe AE, he or she will be withdrawn from the study and referred to their general practitioner or hospital emergency department depending on the nature of the event.

Dissemination

The outcomes of the study will be published in peer-reviewed international scientific journals in accordance with the CONSORT 2010 Statement. The outcomes will be published regardless of whether they are positive, negative, or inconclusive. A written lay summary of the study outcomes will be available for the participants on request. The study outcomes will also be presented as posters or oral presentations at national and international meetings and congresses.

Analyses

All data recordings will be obtained electronically through TSD and analysed by a statistician, blinded for the participant’s group allocation. Baseline demographic and clinical characteristics will be recorded at the 1st chiropractic visit and be tabulated as means and standard deviations (SD) for continuous variables, and proportions and percentages for categorical variables. Primary and secondary end-points will be presented using suitable descriptive statistics in each group and for each time point. The comparison of groups will be performed by a linear mixed model with fixed effects for time, group and interaction between the two. Random effects for participants nested within chiropractors will be included. As primary analysis, change in primary end-point from baseline to day 14 will be compared between group 1 and group 2, group 1 and group 3, and group 1 and group 4. As secondary analyses, comparisons of trend from baseline through follow-up between group 1 and group 2, group 1 and group 3, group 1 and group 4, will be performed for primary and secondary end-points. Moreover, stratified analyses will be performed by estimating the same model with additional fixed effects for sex and age and the interactions between those two and the group and time variables.

Data recording

Onset, numbers of days, duration and neck pain intensity will be recorded at the first chiropractic visit (baseline) prior to randomization, and at 1, 4-, 8-, 12- and 24-weeks post-treatment. Sick leave due to the neck pain episode or for any other reasons will also be monitored throughout the study period.

Additionally, days, intensity and duration of neck pain will be measured every day for 14 days. The specific wording of these three questions will be.

1. "On average, how strong was your neck pain today, on an NRS scale from 0 to 10, where 0 is no pain and 10 is unbearable pain?"
2. “At worst, how strong was your neck pain today, on an NRS scale from 0 to 10, where 0 is no pain and 10 is unbearable pain?"
3. "How many hours did you have neck pain during the past 24 hours?”

RAND-12 and Neck Disability Index questionnaires will be recorded at 1st chiropractic visit (baseline) prior to randomization, at day 14 and at follow-up weeks 12 and 24 (43, 44).

Schedule of activities/events

Baseline

Chiropractor demographics

Inclusion/exclusion criteria
Patients' baseline demographics
Patients' treatment expectations
Chiropractor treatment expectations

**TREATMENT**	Baseline	Day 2	Day 3	Day 4	Day 5	Day 6	Day 7
Treatment activities							
CSMT/Sham CSMT	3 treatment sessions						
Blinding	3 times on the day for CSMT/sham CSMT						
Ibuprofen/placebo medicine	X	X	X	X	X	X	X
Adverse events		X	X	X	X	X	X
Blinding	X	X	X	X	X	X	X
Recordings of events							
Pain intensity	X	X	X	X	X	X	X
Pain duration	X	X	X	X	X	X	X
RAND-12	X						
Neck Disability Index	X						
Patients expectation	X						
Chiropractors expectation	X						

	Day 8	Day 9	Day 10	Day 11	Day 12	Day 13	Day 14
Treatment activities							
CSMT/Sham CSMT	2 treatment sessions						
Blinding	2 times on the day for CSMT/sham CSMT						
Ibuprofen/placebo medicine	X	X	X	X	X		
Treatment satisfaction							X
Adverse events	X	X	X	X	X	X	X
Blinding	X	X	X	X	X	X	X
Recordings of events							
Pain intensity	X	X	X	X	X	X	X
Pain duration	X	X	X	X	X	X	X
RAND-12							X
Neck Disability Index							X
Satisfaction							X

**POSTTREATMENT**	Week 1 (Day 7)	Week 4 (Day 28)	Week 8 (Day 56)	Week 12 (Day 84)	Week 24 (Day 168)
Recordings of events					
Pain intensity	X	X	X	X	X
Pain duration	X	X	X	X	X
RAND-12				X	X
Neck Disability Index				X	X
Manual treatment	X	X	X	X	X
Medicin comsumption	X	X	X	X	X

<u>Objective</u>
<u>Primary objective</u>
Investigate efficacy of CSMT and NSAIDs on the intensity of acute neck pain in the treatment period.

<u>Secondary objective</u>
Investigate efficacy of CSMT and NSAIDs on the intensity of acute neck pain in the follow-up period.
Investigate efficacy of CSMT and NSAIDs on neck pain days and duration at different time points.

Investigate efficacy of CSMT and NSAIDs on impact on health and effect on neck disability.

Primary end-point

The primary end-point will be defined as the mean pain intensity on a NRS 0-10. The primary analysis will assess the difference in mean pain intensity change from baseline to day 14 after baseline between group 1 and group 2, group 1 and group 3, and group 1 and group 4.

Secondary end-points

1. Mean pain intensity (NRS 0-10) change from baseline to day 2, 3, 4, 5, 6, 7, 8, 9, 10, 11, 12, 13 in the treatment period, and from baseline to day 7, 28, 56, 84 and 168 post-treatment, respectively, and comparison between group 1 and group 2, group 1 and group 3, and group 1 and group 4.
2. Mean duration (hours) of neck pain change from baseline to day 2, 3, 4, 5, 6, 7, 8, 9, 10, 11, 12, 13 and 14 after baseline, day 7, 28, 56, 84 and 168 post-treatment, respectively, and comparison between group 1 and group 2, group 1 and group 3, and group 1 and group 4.
3. Mean number of days with neck pain per week from the treatment period (14 days) to the periods; day 1-7, 22-28, 50-56, 78-84 and 162-168 post-treatment, respectively, and comparison between group 1 and group 2, group 1 and group 3, and group 1 and group 4.
4. Proportions of participants with mean daily pain intensity reduction of ≥50%, ≥75% and 100% from baseline to day 2, 3, 4, 5, 6, 7, 8, 9, 10, 11, 12, 13 and 14 in the treatment period, and from baseline to day 7, 28, 56, 84 and 168 post-treatment, respectively, and comparison between group 1 and group 2, group 1 and group 3, and group 1 and group 4.
5. Proportions of participants with mean duration (hours) reduction of ≥50%, ≥75% and 100 from baseline to day 2, 3, 4, 5, 6, 7, 8, 9, 10, 11, 12, 13 and 14 in the treatment period, and from baseline to day 7, 28, 56, 84 and 168 post-treatment, respectively, and comparison between group 1 and group 2, group 1 and group 3, and group 1 and group 4.
6. Proportions of participants with mean reduction of number of days with neck pain per week of ≥50%, ≥75% and 100% from the 14 days treatment period, to the periods day 1-7, 22-28, 50-56, 78-84 and 162-168 post-treatment, respectively, and comparison between group 1 and group 2, group 1 and group 3, and group 1 and group 4.
7. Improvement in RAND-12 score from baseline to day 14 after baseline, day 84 and 168 post-treatment, respectively, comparison between group 1 and group 2, group 1 and group 3, and group 1 and group 4.
8. Improvement in Neck Disability Index score from baseline to day 14 after baseline, day 84 and 168 post-treatment, respectively, and comparison between group 1 and group 2, group 1 and group 3, and group 1 and group 4.
9. AE analysis of group 1, group 2, group 3 and group 4, and comparison between group 1 and group 2, group 1 and group 3, and group 1 and group 4.
10. Analysis of patients' blinding on NRS 0-10 in relation to receiving real CSMT (0 absolutely unsure and 10 absolutely sure that real CSMT was received), irrespectively, whether the patient receive sham or real CSMT, analysis of group 1 and group 2 and comparison between group 1 and group 2.
11. Analysis of patients' blinding on NRS 0-10 in relation to receiving ibuprofen (0 absolutely unsure and 10 absolutely sure that ibuprofen was received), irrespectively, whether the patient receive ibuprofen or placebo medicine, analysis of group 1 and group 2 and comparison between group 1 and group 2.
12. Analysis of patients' and chiropractors' expectations to treatment efficacy on NRS 0-10 (0 no expectation of treatment efficacy and 10 the highest possible expectation to treatment efficacy).
13. Analysis of patients' satisfaction to treatment efficacy on NRS 0-10 (0 no satisfaction at all, and 10 the highest possible satisfaction).

Blinding

After each intervention, the participants will complete a de-blinding questionnaire (34). The included chiropractors will complete the same questionnaire post-treatment.

Adverse events
All AEs will be recorded after each intervention in all four groups in accordance with the CONSORT recommendations (45, 46).

Serious adverse events and suspected unexpected serious adverse reaction
Participants that experience serious adverse event due to the CSMT can contact the chiropractor that applied the treatment (contact details are provided in the list of chiropractors) OR PhD student Anna Allen-Unhammer, Institutt for helse og samfunn, Universitet i Oslo, on mobil phone +47 928 70 783, Participants that experience serious adverse events due to medication can contact PhD student Anna Allen-Unhammer, Institutt for helse og samfunn, Universitet i Oslo, on mobil phone +47 928 70 783, whom will have the documents so it is possible to established the precise medication the patient had received (unblinding/decoding).
SUSARs are reported to Statens Legemiddelverk on the CIOMS reporting form (meldeskjema).
For further details see delegeringslogg.

Expectation
Prior to the 1st intervention session, all participants and chiropractors will complete a simple expectation questionnaire.

Satisfaction
After the last intervention, all participants will complete a simple satisfaction questionnaire.

Statistical power
Power calculation will be performed for primary end-point, average pain intensity measured by NRS. We expect the average NRS score of five at baseline, with standard deviation (SD, representing individual variations) equal to 1 in all groups. A reduction in pain intensity of 60% from baseline to day 14 after baseline is expected in group 1, 40% reduction is expected in group 2 and group 3, while a reduction of 20% is expected in group 4. We assume the same SD of 1 at day 14 after baseline. Due to three comparisons (group 1 vs. 2, group 1 vs. 3, and group 1 vs. 4) we will reduce the nominal significance level of 0.05 to 0.017 to account for multiple testing. The sample size necessary to show a statistically significant difference between the groups regarding the reduction in NRS score from baseline to day 14 after baseline with the power of 80%, was estimated to be 43 patients in group 1, 2 and 3 and 12, in group 4. Since we are going to conduct a multi-centre practice-based study including ≥20 chiropractors, an intra-chiropractor correlation, or cluster effect, are likely to be present in our data. We assume such a cluster effect to be about 25%, a rather conservative estimate. After adjustment for cluster effect, 56 patients in group 1, 2 and 3 will have to be included. To maintain blinding throughout the statistical analyses, 56 patients will have to be included in group 4. Some drop-outs are to be expected. In order to maintain as high power as possible, we aim to include 80 patients per group, i.e., 16 patients per chiropractor, partitioned into four groups with four patients randomly assigned to each group. This means a difference of 1 on the NRS (0-10) can be detected which is held to be within the minimal clinically important difference (47).

Participants, organization and collaborations

Michael Bjørn Russell – project leader and co-supervisor, professor of neurology, consultant neurologist, PhD, DrMedSci
Aleksander Chaibi – main supervisor, PhD, chiropractor (DC), and physiotherapist (PT)
Nina Køpke Vøllestad – co-supervisor, PhD, professor at Faculty of Medicine, Institute of Health and Society, University of Oslo, Norway
Anna Jane Allen-Unhammer (PhD student)
Chiropractors – ≥20 national chiropractors (multi-centre).

Registration and publication of trial protocol

The trial protocol will be registered at ClinicalTrials.gov and published in a scientific journal, and Legemiddelverket will register the trial protocol in EU Clinical Trials Register (EUCTR) before inclusion of patients.

Budget

Stiftelsen Dam has granted funds for the PhD student’s salary. An application has been sent to Helse Sør-Øst for supplementary financing, i.e., cost for medication. The project leader and co-supervisors hold academic positions and the main supervisor will be affiliated with the HELSAM, UiO.

Progression plan

12 months: Chiropractor training and recruitment, implementation of data recordings, courses.
12 months: Clinical monitoring data collection, analysis, drafting the scientific papers, courses.
31.12.2023 END OF CLINICAL TRIAL
12 months: Drafting additional papers, additional PhD courses, and PhD thesis.

Publication plan

The PhD project stipulates a minimum of three papers published in high impact scientific peer reviewed medical journals.
i) Chiropractic spinal manipulative therapy for acute neck pain: a study protocol of a 4-armed, randomized placebo-controlled trial.
ii) Chiropractic spinal manipulative therapy for acute neck pain: a 4-armed, randomized placebo-controlled trial.
iii) Validation of sham manipulation (placebo) in a multi-centre practice-based chiropractic manual-therapy randomized controlled trial.
iv) Adverse events in chiropractic spinal manipulative therapy vs. ibuprofen in patients with acute neck pain. A randomized, semi double-blind, multi-centre, placebo-controlled clinical trial.

Plan for activities, visibility and dissemination

The chiropractic PhD student and the PostDoc will participate once or twice per year at National and International meetings and congresses of relevance, and present results. Data will be published in high impact medical journals, in order to diminish the barrier and allow for better professional distribution between the medical and the chiropractic and/or manual-therapy professions.

Plan for implementation and visibility

Our experience with a previous chiropractic RCT for migraine is that it had major interest right after publication (27). This was also true for our review papers (48-50), prior to the RCT results being published. This was probably due to publication in high impact medical journals and free access to the e-publication. We will pursue the same strategy for this project.

User involvement

The project will be associated with users from Ryggforeningen i Norge, Norway. They will function as discussion partners. When the results of the study are available, they will assist in disseminating the results to patients, patient organizations and healthcare professionals who treat this patient group. This will be conducted through articles in scientific high impact journals, lectures, member magazines and other relevant publications. Ryggforeningen i Norge may also be a driving force to implement the new knowledge from the project quickly.

Innovative and scientific value

This 4-armed manual-therapy RCT will highlight and validate chiropractic manual-therapy and medication for participants with acute neck pain. The study design protects against the many warned biases in manual-therapy RCT. This is to our knowledge the first RCT to include both a manual sham placebo arm as well as a pharmacological placebo arm. The introduction of a validated manual sham placebo arm is a major innovative improvement which lifts manual-therapy RCTs to the level of

pharmacological RCTs and makes it possible to evaluate true net effect (34). Studies with unimodal approaches isolate (statistically) the individual effects of SMT better than multimodal approaches do. Assessing the effect of multimodal programs is problematic, because it is difficult to isolate the impact of a specific intervention. Although most cases of acute neck pain, regardless of whether or not they are radicular in nature, will resolve within three months, a substantial proportion of people will continue to experience low-grade symptoms or frequent recurrences (5). Thus, the study has rationalized a triangle of importance for stakeholders between patients'-, health care system- and research benefits.

Limitations and strengths

Limitations

All clinical studies of certain durations have a risk of drop-out. However, this is minimized through the relatively short intervention- and follow-up period.

There is a recruitment risk since patients might have an expectation towards chiropractic treatment and not medication, when seeking care. This will be resolved by adding more recruiting chiropractic clinics.

Risk of unsuccessful blinding in the CSMT sham manipulation group due to multicentre study design and ≥20 chiropractors. Nevertheless, the manual placebo has been evaluated in >30 international clinical studies with concealed placebo groups.

Although this is very difficult to control for, we need to acknowledge that known contextual factors might be present and influence the placebo response.

The study will be the first 4-armed manual-therapy and medication RCT that will assess the efficacy of four different intervention groups; 1) CSMT, 2) CSMT sham manipulation, 3) Ibuprofen medication and 4) placebo medication, for patients with acute neck pain.

To our knowledge, this is the first manual-therapy RCT to include two placebo groups.

There is a strong external validity, which improves the generalizability.

The RCT has the potential to provide evidence-based non-pharmacological and pharmacological treatment option for patients with acute neck pain.

Delegation log
(Delegereingslogg)

1. Chiropractor recruit and randomize participants in the study.
2. Chiropractor provide participants allocated to medicine with medication.
3. Chiropractor provide manual therapy to participants allocated to manual therapy.
4. Chiropractor receive information and manage serious adverse event due to manual therapy.
5. PhD student receive information and manage serious adverse event due to medication along with the project leader (MD).
6. PhD student fill in SUSAR and sent it to SLV.

______________________________	______________________________
Chiropractor signature and date	PhD student signature and date
______________________________	Anne Jane Allen-Unhammer
Name capital letters	Name capital letters

References

[1] Vos T, Flaxman AD, Naghavi M, Lozano R, Michaud C, Ezzati M, et al. Years lived with disability (YLDs) for 1160 sequelae of 289 diseases and injuries 1990-2010: a systematic analysis for the Global Burden of Disease Study 2010. Lancet. 2012;380:2163-96.

[2] Global, regional, and national incidence, prevalence, and years lived with disability for 310 diseases and injuries, 1990-2015: a systematic analysis for the Global Burden of Disease Study 2015. Lancet. 2016;388:1545-602.

[3] Kamper SJ, Henschke N, Hestbaek L, Dunn KM, Williams CM. Musculoskeletal pain in children and adolescents. Brazilian journal of physical therapy. 2016;20:275-84.

[4] Hoy D, March L, Woolf A, Blyth F, Brooks P, Smith E, et al. The global burden of neck pain: estimates from the global burden of disease 2010 study. Annals of the rheumatic diseases. 2014;73:1309-15.

[5] Cohen SP, Hooten WM. Advances in the diagnosis and management of neck pain. BMJ. 2017;358:j3221.

[6] Dieleman JL, Baral R, Birger M, Bui AL, Bulchis A, Chapin A, et al. US Spending on Personal Health Care and Public Health, 1996-2013. Jama. 2016;316:2627-46.

[7] Lærum E, Brage S, Ihlebæk C, Johnsen K, Natvig B, Aas E. Et muskel- og skjelettregnskap Forekomst og kostnader knyttet til skader, sykdommer og plager i muskel- og skjelettsystemet. www.formi.no: Muskel og Skjelett Tiåret (MST) v/ FORMI, Klinikk for kirurgi og nevrofag, Oslo universitetssykehus – Ullevål 2013:92.

[8] Skogli E, Theie MG, Stokke OM, Lind LH. Muskel- og skjelettsykdom i Norge: Rammer flest - Koster mest. Vurderinger av tiltak for å redusere samfunnskostnadene. Vol 31, 2019:56.

[9] Dikkers MF, Westerman MJ, Rubinstein SM, van Tulder MW, Anema JR. Why Neck Pain Patients Are Not Referred to Manual Therapy: A Qualitative Study among Dutch Primary Care Stakeholders. PloS one. 2016;11:e0157465.

[10] Roelofs PD, Deyo RA, Koes BW, Scholten RJ, van Tulder MW. Nonsteroidal anti-inflammatory drugs for low back pain: an updated Cochrane review. Spine. 2008;33:1766-74.

[11] Predel HG, Ebel-Bitoun C, Lange R, Weiser T. A randomized, placebo- and active-controlled, multi-country, multi-center parallel group trial to evaluate the efficacy and safety of a fixed-dose combination of 400 mg ibuprofen and 100 mg caffeine compared with ibuprofen 400 mg and placebo in patients with acute lower back or neck pain. Journal of pain research. 2019;12:2771-83.

[12] Carroll LJ, Hogg-Johnson S, van der Velde G, Haldeman S, Holm LW, Carragee EJ, et al. Course and prognostic factors for neck pain in the general population: results of the Bone and Joint Decade 2000-2010 Task Force on Neck Pain and Its Associated Disorders. Spine. 2008;33:S75-82.

[13] Hogg-Johnson S, van der Velde G, Carroll LJ, Holm LW, Cassidy JD, Guzman J, et al. The burden and determinants of neck pain in the general population: results of the Bone and Joint Decade 2000-2010 Task Force on Neck Pain and Its Associated Disorders. Spine. 2008;33:S39-51.

[14] Furlan AD, Yazdi F, Tsertsvadze A, Gross A, Van Tulder M, Santaguida L, et al. A systematic review and meta-analysis of efficacy, cost-effectiveness, and safety of selected complementary and alternative medicine for neck and low-back pain. Evidence-based complementary and alternative medicine : eCAM. 2012;2012:953139.

[15] Gross A, Langevin P, Burnie SJ, Bedard-Brochu MS, Empey B, Dugas E, et al. Manipulation and mobilisation for neck pain contrasted against an inactive control or another active treatment. The Cochrane database of systematic reviews. 2015;9:Cd004249.

[16] Kvammen OC, Leboeuf-Yde C. The chiropractic profession in Norway 2011. Chiropractic & manual therapies. 2014;22:44.

[17] Esposito S, Philipson S. Spinal adjustment technique the chiropractic art. Alexandria: Craft Printing, 2005.

[18] McLain RF, Pickar JG. Mechanoreceptor endings in human thoracic and lumbar facet joints. Spine (Phila Pa 1976). 1998;23:168-73.
[19] Vernon H. Qualitative review of studies of manipulation-induced hypoalgesia. J Manipulative Physiol Ther. 2000;23:134-8.
[20] Vicenzino B, Paungmali A, Buratowski S, Wright A. Specific manipulative therapy treatment for chronic lateral epicondylalgia produces uniquely characteristic hypoalgesia. Man Ther. 2001;6:205-12.
[21] Boal RW, Gillette RG. Central neuronal plasticity, low back pain and spinal manipulative therapy. J Manipulative Physiol Ther. 2004;27:314-26.
[22] Bialosky JE, Bishop MD, Price DD, Robinson ME, George SZ. The mechanisms of manual therapy in the treatment of musculoskeletal pain: a comprehensive model. Manual therapy. 2009;14:531-8.
[23] De Camargo VM, Alburquerque-Sendin F, Berzin F, Stefanelli VC, de Souza DP, Fernandez-de-las-Penas C. Immediate effects on electromyographic activity and pressure pain thresholds after a cervical manipulation in mechanical neck pain: a randomized controlled trial. J Manipulative Physiol Ther. 2011;34:211-20.
[24] Newell D, Lothe LR, Raven TJL. Contextually Aided Recovery (CARe): a scientific theory for innate healing. Chiropractic & manual therapies. 2017;25:6.
[25] Turner JA, Deyo RA, Loeser JD, M. VK, Fordyce WE. The importance of placebo effects in pain treatment and research. JAMA. 1994;271:1609-14.
[26] Hancock MJ, Maher CG, Latimer J, McAuley JH. Selecting an appropriate placebo for a trial of spinal manipulative therapy. Aust J Physiother. 2006;52:135-8.
[27] Chaibi A, Benth JS, Tuchin P, Russell MB. Chiropractic spinal manipulative therapy for migraine: a three-armed, single-blinded, placebo, randomized controlled trial. Eur J Neurol. 2017;24:143-53.
[28] Chaibi A, Knackstedt H, Tuchin PJ, Russell MB. Chiropractic spinal manipulative therapy for cervicogenic headache: a single-blinded, placebo, randomized controlled trial. BMC research notes. 2017;10:310.

[29] Aspinall SL, Jacques A, Leboeuf-Yde C, Etherington SJ, Walker BF. No difference in pressure pain threshold and temporal summation after lumbar spinal manipulation compared to sham: A randomised controlled trial in adults with low back pain. Musculoskeletal science & practice. 2019;43:18-25.
[30] Picchiottino M, Honoré M, Leboeuf-Yde C, Gagey O, Cottin F, Hallman DM. The effect of a single spinal manipulation on cardiovascular autonomic activity and the relationship to pressure pain threshold: a randomized, cross-over, sham-controlled trial. Chiropractic & manual therapies. 2020;28:7.
[31] Honoré M, Picchiottino M, Wedderkopp N, Leboeuf-Yde C, Gagey O. What is the effect of spinal manipulation on the pressure pain threshold in young, asymptomatic subjects? A randomized placebo-controlled trial, with a cross-over design. Chiropractic & manual therapies. 2020;28:6.
[32] Holt K, Niazi IK, Amjad I, Kumari N, Rashid U, Duehr J, et al. The Effects of 4 Weeks of Chiropractic Spinal Adjustments on Motor Function in People with Stroke: A Randomized Controlled Trial. Brain sciences. 2021;11.
[33] Lynge S, Dissing KB, Vach W, Christensen HW, Hestbaek L. Effectiveness of chiropractic manipulation versus sham manipulation for recurrent headaches in children aged 7-14 years - a randomised clinical trial. Chiropractic & manual therapies. 2021;29:1.
[34] Chaibi A, Saltyte Benth J, Bjorn Russell M. Validation of Placebo in a Manual Therapy Randomized Controlled Trial. Scientific reports. 2015;5:11774.
[35] Guzman J, Haldeman S, Carroll LJ, Carragee EJ, Hurwitz EL, Peloso P, et al. Clinical practice implications of the Bone and Joint Decade 2000-2010 Task Force on Neck Pain and Its Associated Disorders: from concepts and findings to recommendations. Spine. 2008;33:S199-213.

[36] Uitvlugt G, Indenbaum S. Clinical assessment of atlantoaxial instability using the Sharp-Purser test. Arthritis and rheumatism. 1988;31:918-22.

[37] Chaibi A, Russell MB. A risk-benefit assessment strategy to exclude cervical artery dissection in spinal manual-therapy: a comprehensive review. Annals of medicine. 2019:1-10.

[38] Cooperstein R, Gleberson BJ. Technique systems in chiropractic. 1 ed. New York: Churchill Livingston, 2004.

[39] Chaibi A, Saltyte Benth J, Tuchin PJ, Russell MB. Chiropractic spinal manipulative therapy for migraine: a study protocol of a single-blinded placebo-controlled randomised clinical trial. BMJ open. 2015;5:e008095.

[40] Chaibi A, Benth JS, Tuchin PJ, Russell MB. Chiropractic spinal manipulative therapy for cervicogenic headache: a study protocol of a single-blinded placebo-controlled randomized clinical trial. SpringerPlus. 2015;4:779.

[41] Foster NE, Anema JR, Cherkin D, Chou R, Cohen SP, Gross DP, et al. Prevention and treatment of low back pain: evidence, challenges, and promising directions. Lancet. 2018.

[42] Chou R, Cote P, Randhawa K, Torres P, Yu H, Nordin M, et al. The Global Spine Care Initiative: applying evidence-based guidelines on the non-invasive management of back and neck pain to low- and middle-income communities. European spine journal : official publication of the European Spine Society, the European Spinal Deformity Society, and the European Section of the Cervical Spine Research Society. 2018.

[43] Vernon H, Mior S. The Neck Disability Index: a study of reliability and validity. J Manipulative Physiol Ther. 1991;14:409-15.

[44] Feeny D, Farris K, Cote I, Johnson JA, Tsuyuki RT, Eng K. A cohort study found the RAND-12 and Health Utilities Index Mark 3 demonstrated construct validity in high-risk primary care patients. Journal of clinical epidemiology. 2005;58:138-41.

[45] Leboeuf-Yde C, Hennius B, Rudberg E, Leufvenmark P, Thunman M. Side effects of chiropractic treatment: a prospective study. J Manipulative Physiol Ther. 1997;20:511-5.

[46] Moher D, Hopewell S, Schulz KF, Montori V, Gotzsche PC, Devereaux PJ, et al. CONSORT 2010 explanation and elaboration: updated guidelines for reporting parallel group randomised trials. BMJ. 2010;340:c869.

[47] Olsen MF, Bjerre E, Hansen MD, Hilden J, Landler NE, Tendal B, Hróbjartsson A. Pain relief that matters to patients: systematic review of empirical studies assessing the minimum clinically important difference in acute pain. BMC medicine. 2017;15:35.

[48] Chaibi A, Tuchin PJ, Russell MB. Manual therapies for migraine: a systematic review. J Headache Pain. 2011;12:127-33.

[49] Chaibi A, Russell MB. Manual therapies for cervicogenic headache: a systematic review. J Headache and Pain. 2012;13:351-9.

[50] Chaibi A, Russell MB. Manual therapies for primary chronic headaches: a systematic review of randomized controlled trials. J Headache Pain. 2014;15:67.
